# Differential levels of gene expression and molecular mechanisms between red maple (*Acer rubrum*) genotypes resistant and susceptible to nickel toxicity revealed by transcriptome analysis

**DOI:** 10.1002/ece3.4045

**Published:** 2018-04-19

**Authors:** Kabwe Nkongolo, Gabriel Theriault, Paul Michael

**Affiliations:** ^1^ Biomolecular Sciences Program Laurentian University Sudbury ON Canada; ^2^ Department of Biology Laurentian University Sudbury ON Canada

**Keywords:** *Acer rubrum*, candidate genes, differential expression, nickel resistance, transcriptome

## Abstract

Knowledge of regulation of genes associated with metal resistance in higher plants is very limited. Many plant species have developed different genetic mechanisms and metabolic pathways to cope with metal toxicity. The main objectives of this study were to 1) assess gene expression dynamics of *A. rubrum* in response to nickel (Ni) stress and 2) describe gene function based on ontology. Certified *A. rubrum* genotypes were treated with 1,600 mg of Ni per 1 Kg of soil corresponding to a soil total nickel content in a metal‐contaminated region in Ontario, Canada. Nickel resistant and susceptible genotypes were selected and used for transcriptome analysis. Overall, 223,610,443 bases were generated. Trinity reads were assembled to trinity transcripts. The transcripts were mapped to protein sequences and after quality controls and appropriate trimmings, 66,783 annotated transcripts were selected as expressed among the libraries. The study reveals that nickel treatment at a high dose of 1,600 mg/kg triggers regulation of several genes. When nickel‐resistant genotypes were compared to water controls, 6,263 genes were upregulated and 3,142 were downregulated. These values were 3,308 and 2,176, respectively, when susceptible genotypes were compared to water control. The coping mechanism of *A. rubrum* to Ni toxicity was elucidated. Upregulation of genes associated with transport in cytosol was prevalent in resistant genotypes compared to controls while upregulation of genes associated with translation in the ribosome was higher in susceptible genotypes when compared to water. The analysis revealed no major gene associated with Ni resistance in *A. rubrum*. Overall, the results of this study suggest that the genetic mechanism controlling the resistance of this species to nickel is controlled by genes with limited expression. The subtle differences between resistant and susceptible genotypes in gene regulation were detected using water‐treated genotypes as references.

## 1 INTRODUCTION

Plants deal with toxic levels of metals such as nickel (Ni) using different mechanisms. Ni is a transition metal that is found in natural soils at low concentrations. An excess of Ni ions in the soil results in the disorder of cell membrane functions, inhibition of cell division in the root system, and a decrease of nontolerant plant growth (Yadav, 2010). It can also increase the concentration of hydroxyl radicals, superoxide anions, nitric oxide, and hydrogen peroxide (Bhalerao, Sharma, & Poojari, 2015; Boominathan & Doran, 2002; Rao & Sresty, 2000; Stohs et al., 2001). Metal ions that accumulate in plant systems usually disturb cellular ion homeostasis and can generate (directly or indirectly) reactive oxygen species (ROS) which may cause oxidative stress. The toxicity of ROS can lead to the destruction of DNA structure and enhance the oxidation of lipids and proteins.

Nickel has a complex chemistry which complicates the decryption of its toxicity mechanisms in plants (Bhalerao et al., 2015). It does not directly induce the production of ROS as it is not a redox‐active metal. Its role in ROS production is an indirect one by inhibiting the function of several antioxidant enzymes which include ascorbate peroxidase (APX), catalase (CAT), glutathione peroxidase (GSH‐Px), glutathione reductase (GR), guaiacol peroxidase (GOPX), peroxidase (POD), and superoxide dismutase (SOD) (Bhalerao et al., 2015; Freeman et al., 2004; Gomes‐Junior et al., 2006; Pandey & Sharma, 2002).

Nickel translocation and toxicity in hardwood species has been recently a focus of a few reports. It has been demonstrated that white birch (*Betula papyrifera*), trembling aspen (*Populus tremuloides*), and red oak (*Quercus rubra*) accumulate nickel in leaves. Therefore, they are classified as nickel accumulators (Mehes‐Smith & Nkongolo, 2015; Theriault, Michael, & Nkongolo, 2016a; Theriault, Nkongolo, & Michael, 2014; Theriault et al., 2013; Tran et al., 2014). Red maple (*Acer rubrum*) on the other hand does not accumulate nickel in its tissues (the amount of bioavailable nickel in the soil is higher than the total nickel in roots). The translocation of nickel from roots to aerial parts is also very small. This species can be therefore classified as a nickel avoider (Kalubi, Mehes‐Smith, & Omri, 2016; Kalubi et al., 2015). *A. sacharinium*, a close relative of *A. rubrum,* stores Ni in its roots with limited translocation to other plant parts. It is classified as a Ni excluder (Nkongolo, Narendrula‐Kotha, Kalubi, Rainville, & Michael, 2017). We anticipated that genetic mechanisms and metabolic pathways involved in coping with nickel toxicity in these hardwood species, with distinct strategies in dealing with this element in their system, would be different.

Most resistant plants that accumulate metals in their tissues have developed a detoxification mechanism that involves phytochelatin (PC). These tri‐peptides are synthesized from reduced glatathione (GSH). GSH conjugates with heavy metal molecules through glutathione S‐transferase during detoxification processes. Many studies have demonstrated that genes controlling glyoxalases, phytochelatin synthase, glutathione reductase, serine acetyltransferase, ATP sulfuylase, cystathionine synthase, glutathione synthetase, and γ‐glutamycylcysteine synthetase are candidates for providing metal tolerance by regulating GSH and PCs levels in accumulator plants (Yadav, 2010). Our understanding of genetic mechanisms involved in metal avoidance in plant species such as *A. rubrum* is vague.

The main objectives of this study were to (1) assess gene expression dynamics of *A. rubrum* in response to nickel stress and (2) describe gene function based on ontology. The study provides the first description of molecular and biological processes involved in Ni resistance in *A. rubrum*.

## 2 MATERIALS AND METHODS

### 2.1 Nickel treatment

*Acer rubrum* seeds were provided by the National Tree Seed Centre, Canadian Forest Services (New Brunswick, Canada). These certified seeds (accession # 2001 1031.0) were collected from Larry Brook, New Brunswick (Canada). Assessment of nickel toxicity is described in Theriault and Nkongolo (2016) and Theriault et al. (2016a). The experimental layout was a completely randomized design with one Ni treatment and two types of control. To assess the genetic resistance of *A. rubrum* genotypes to Ni, 45 six‐month seedlings were treated with 1,600 g of Ni per 1 kg of dry soil using nickel nitrate [Ni(NO_3_)_2_] salts and grown in a growth chamber. This concentration that was used in previous studies corresponds to the level of total nickel in contaminated sites in the mining region of the City of Greater Sudbury. Details of seed germination and seedlings treatment with nickel nitrate are presented in Theriault and Nkongolo (2016) and Theriault et al. (2016a). Genotypes treated with water only were used as the main control. Potassium nitrate (KNO_3_) treatment was used to control for the nitrate effects. The treatment and the controls were replicated 15 times. Damage rating was recorded every two days based on a scale of 1 to 9, 1 =  no visible toxicity symptoms and 9 =  dead plants. Individual plants with a score of 1 to 3 were considered nickel resistant, 4 to 6, moderately resistant, and 7 to 9 susceptible (Theriault & Nkongolo, 2016). Genotypes resistant and susceptible to a soil nickel concentration of 1,600 mg/kg are analyzed in detail. For transcriptome analysis, three Ni resistant and three Ni susceptible genotypes were selected along with three genotypes from each of the controls (water and nitrate controls).

### 2.2 *De novo* transcriptome assembly

Methods for extraction, RNA‐seq libraries, next‐generation sequencing, and *de Novo* transcriptome assembly are detailed in Theriault, Michael, and Nkongolo (2016b). The libraries were quantified using Bioanalyzer 2100 (Agilent Technologies, Santa Clara, CA, USA) and the sequencing was performed on the Illumina HiSeq 2000 sequencing system (Illumina Inc.) at Seq Matic (Fremont California, USA). The RNA‐seq data from all the samples including six nickel‐treated (three resistant and three susceptible), three water‐treated (control), and three nitrate‐treated were used as input for the Trinity program (http:trinityrnaseq.githb.io) to assemble the transcripts. The raw reads were mapped to Trinity assembled transcripts using bowtie (http://bowtie-bio.sourceforge.net/index.shtml), and RSEM (http://deweylab.biostat.wisc.edu/rsem) was used to quantify transcript and expression levels. Additional QC at transcript level was performed, including number of unique transcripts detected, percentage of reads belonging to the top transcripts expressed, normalization for RNA composition, and grouping, and correlation between samples.

If a transcript had a count per million (CPM) value ≥1 in at least two of the samples, we considered it expressed in the experiment and included it for downstream QC analysis. Gene expression was calculated and expressed as Reads Per Kilobase per Million reads mapped (RPKM) (Mortazavi et al., 2008). The count per million (CPM) cutoff was 0.63 based on the average read count of all samples (15.9 million). This CPM cutoff roughly equaled to 10 raw reads in this experiment. A gene with a CPM value >0.63 in at least two samples from the experiment was included for downstream analysis. The raw counts were normalized using the voom method from the R Limma package (http://www.bioconductor.org/packages/release/bioc/html/limma.html) (Law et al., 2014). After normalization, most samples looked similar.

Multidimensional plots were created to view sample relationships. This was performed using R Limma package. We also used the made4 (multivariate analysis of microarrays data using ADE4) program to cluster samples and drew heatmaps based on genes that had variable expression across samples. These variable genes were chosen based on a standard deviation (SD) of expression values larger than 30% of the mean expression value. Genes with a mean logCPM <1 were removed.

For differentially expressed genes, the normalized data were transformed to log2CPM values using the voom method from the R Limma package. A linear model was built for each comparison using the R Limma package, and statistics for differential expression analysis were calculated. The statistical values included log fold change (logFC), *p*‐value, and false discovery rate (FDR). An FDR of 0.05 was used as the standard cutoff (two‐fold change) to determine differentially expressed genes between treatments.

All transcripts were mapped to protein sequences in the UniProt database (http:// http://www.uniprot.org/) and the best match was used to annotate genes and assign gene ontology information. The annotated sequences were run through the GO‐Slim function of the BLAST2GO program to provide a summary known gene functions (Conesa & Götz, 2008). The ontology categories included biological process, cellular components, and molecular function.

## 3 RESULTS

### 3.1 *Acer rubrum* resistance to nickel

The genotypes screened in this study expressed a high level of resistance to Ni. In fact, most seedlings treated with nickel nitrate showed no damage (rating of 1 on the 1 to 9 scale) after two weeks of treatment with the exception of three genotypes that were susceptible (rating of 7). No plant damage from the nitrate control treatment was observed.

### 3.2 Transcription assembly and gene ontology

Several cDNA libraries representing resistant and susceptible *A. rubrum* genotypes along with controls were sequenced using the Illumina HiSeq 2000 high‐throughput platform. Overall, 223,610,443 bases were generated. The average read length was 546.50. The number of transcripts was similar among the different groups (control, resistant, and susceptible). This transcriptome shotgun assembly project has been deposited in the DDBJ/EMBL/GenBank database under the SRA project number SRP098922.

**Figure 1:**
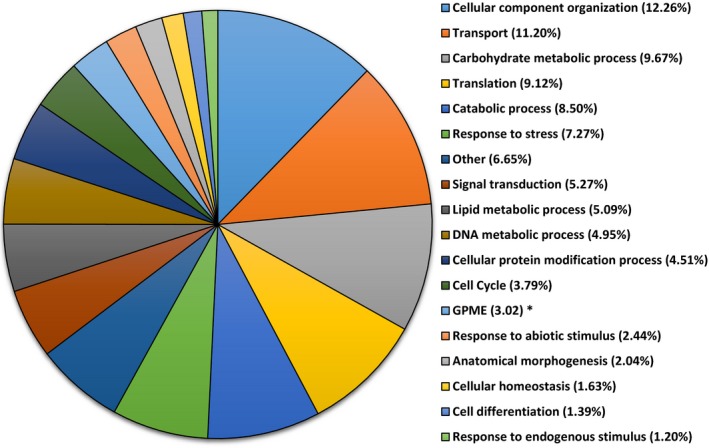
Gene ontology of transcripts from red maple (*Acer rubrum*) control plants (water only). A total of 15,078 transcripts were grouped under biological function using the BLAST2GO software. * GPME stands for Generation of precursor metabolites and energy

**Figure 2:**
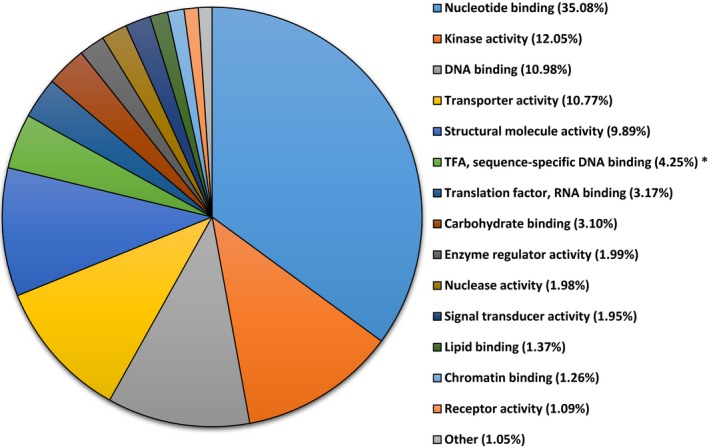
Gene ontology of transcripts from red maple (*Acer rubrum*) control plants (water only). A total of 13,676 transcripts were grouped under molecular function using the BLAST2GO software. * TFA stands for Transcription factor activity

**Figure 3:**
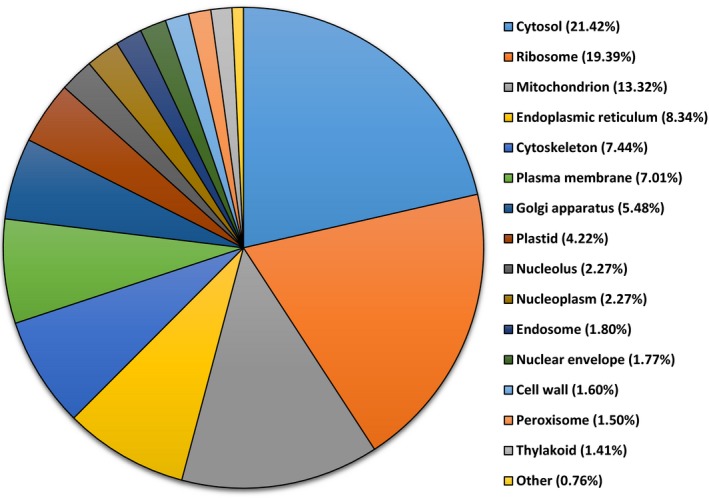
Gene ontology of transcripts from red maple (*Acer rubrum*) control plants (water only). A total of 6,951 transcripts were grouped under cellular compartment using the BLAST2GO software

Transcripts were assigned ontology and grouped by biological process, molecular functions, and cellular components. For biological processes, 58% of the 15,078 transcripts that were assigned ontology were identified under the following categories: cellular component organization (12.26%), transport (11.20%), carbohydrate metabolic process (9.67%), translation (9.12%), catabolic process (8.50%), and response to stress (7.27%) (Figure 1). For molecular functions, 35% of transcripts code for proteins involved in nucleotide binding activities, 12.05% kinase activities, 11% DNA binding, and 10.2% transport activities (Figure 2). For cellular component, 21.4% of the 13,676 transcripts that were assigned ontology were localized in the cytosol, 19.39% in the ribosome, 8.34% in the endoplasmic reticulum, 7.44% in the cytoskeleton, 7% in the plasma membrane, and 5.5% in the Golgi apparatus (Figure 3).

Among the three principal ontologies, most of the expressed transcripts were classified into cellular component organization, transport, carbohydrate metabolic process, translation, catabolic process, and response to stress suggesting that these functional processes play a major role in *A. rubrum* gene activities.

### 3.3 Differential gene expression

Hierarchical clustering can provide good indications of sample and gene relationships. The overall heatmap shows that clustering was good with all the controls (water and nitrate controls were similar to each other). Any differential gene expression was attributed to nickel treatments. Hence, water and nitrate control data were pooled. After normalization, a total of 66,783 transcripts were selected as differentially expressed. Surprisingly, there were no significant differences between resistant and susceptible genotypes at high stringency (FDR <0.05). This means that no major genes driving the resistance to Ni were identified in the *A. rubrum* genotypes analyzed. However, significant differences were observed when resistant genotypes (RG) or susceptible genotypes (SG) were compared to water. When RG were compared to water controls, 6,263 transcripts were upregulated and 3,142 were downregulated. These values were 3,308 and 2,176, respectively, when SG was compared to the same controls.

### 3.4 Pairwise comparison of resistant and susceptible genotypes to water controls

The top upregulated and downregulated transcripts from the differential expression analysis were ranked based on logFC (Tables 1, 2, 3, 4 and Tables S1–S4). The study reveals that the nickel treatment at a high dose of 1,600 mg/kg triggers regulation of several genes. We found that when RG and SG are compared to water, 11% of the top 100 differentially expressed transcripts that were upregulated are shared (Table S5). This value was 25% when the top 100 differentially expressed transcripts that were downregulated in RG and SG were compared to water control (Table S6). Detailed description of the top 25 most upregulated and downregulated transcripts is presented in Tables 1 and 2. There were no particular activities that could be associated with nickel resistance and the highly expressed (or repressed) genes in resistant or susceptible genotypes. But, genes associated with alkaline proteinase were the most upregulated in both RG and SG when compared to water, with logFC values of 11.99 and 10.43, respectively. This means that this transcript (gene) was upregulated 4,068 and 1,380 fold in RG and SG, respectively, when compared to water. Heat maps of comparative gene regulation are illustrated in Figures S1, S2, and S3.

**Table 1:** Top 25 upregulated transcripts in resistant red maple (*Acer rubrum*) genotypes compared to water based on LogFC

Rank	Transcript ID	Plants (RPKM)						LogFC	Adj. *p* Value	Description
		Res. 1	Res. 2	Res. 3	Water 1	Water 2	Water 3			
1	TRINITY_DN426651_c2_g1	159.05	366.63	365.78	0.00	0.00	0.00	11.99	.00208	Alkaline proteinase
2	TRINITY_DN420778_c2_g4	78.36	142.49	169.90	0.00	0.00	0.04	11.87	.00209	Proteinase T
3	TRINITY_DN425833_c0_g1	26.79	58.46	56.29	0.00	0.00	0.00	11.64	.00208	Predicted protein
4	TRINITY_DN428667_c1_g1	64.11	146.03	68.19	0.00	0.00	0.00	11.54	.00208	GRF domain class transcription factor
5	TRINITY_DN437630_c5_g2	75.50	329.35	204.28	0.00	0.00	0.00	10.81	.00228	Ribosomal protein S30
6	TRINITY_DN425283_c1_g1	538.38	925.45	985.42	0.00	0.00	18.17	10.80	.01728	CFEM‐domain‐containing protein
7	TRINITY_DN422756_c0_g1	154.07	587.86	398.80	0.00	0.00	0.00	10.75	.00208	Elongation factor 1‐alpha
8	TRINITY_DN402821_c0_g1	17.72	45.29	22.73	0.00	0.00	0.00	10.71	.00208	Glycoside hydrolase family 54 protein
9	TRINITY_DN431004_c1_g4	83.01	492.12	298.61	0.00	0.00	0.00	10.70	.00327	Ribosomal protein
10	TRINITY_DN428052_c1_g1	43.29	244.97	173.21	0.00	0.00	0.41	10.60	.04443	Kelch repeat protein
11	TRINITY_DN373295_c0_g2	61.42	326.93	157.24	0.00	0.00	0.00	10.46	.00254	Predicted protein
12	TRINITY_DN432608_c1_g1	53.48	249.63	177.29	0.00	0.00	0.37	10.45	.02171	CFEM‐domain‐containing protein
13	TRINITY_DN426185_c1_g2	11.53	78.81	45.91	0.00	0.00	0.00	10.43	.00362	GPI anchored serine‐rich protein
14	TRINITY_DN389300_c0_g2	21.12	171.08	39.68	0.00	0.00	0.00	10.31	.00418	Endo‐1,4‐beta‐glucanase
15	TRINITY_DN436844_c0_g1	23.67	160.07	48.99	0.00	0.00	0.00	10.30	.00315	Predicted protein
16	TRINITY_DN435092_c1_g1	14.67	94.06	20.07	0.00	0.00	0.09	10.28	.02697	WGS project CABT00000000 data
17	TRINITY_DN432457_c1_g3	18.66	90.77	61.94	0.00	0.00	0.00	10.26	.00327	Putative ubiquitin conjugating enzyme (UbcD)
18	TRINITY_DN418791_c0_g1	49.13	203.79	60.55	0.00	0.00	0.00	10.19	.00210	Predicted protein
19	TRINITY_DN426651_c1_g1	57.06	143.20	136.41	0.00	0.00	0.00	10.18	.00208	Alkaline proteinase
20	TRINITY_DN436706_c1_g2	18.57	85.44	39.26	0.00	0.00	0.00	10.17	.00209	Probable ribosomal protein L9.e.c14
21	TRINITY_DN405758_c1_g1	7.43	104.65	19.32	0.00	0.00	0.00	10.17	.00775	Mannanase
22	TRINITY_DN439596_c1_g1	106.96	36.90	36.58	0.00	0.00	0.00	10.15	.00208	Putative growth factor independence
23	TRINITY_DN424010_c0_g2	4.85	34.24	23.09	0.00	0.00	0.00	10.14	.00368	Prenylated Rab acceptor 1
24	TRINITY_DN431808_c3_g3	46.13	206.90	115.58	0.00	0.00	0.00	10.14	.00210	Chromosome 4, complete genome
25	TRINITY_DN447378_c2_g1	40.06	27.74	79.39	0.00	0.00	0.00	10.13	.00209	Glycosyltransferase

**Table 2:** Top 25 downregulated transcripts in resistant red maple (*Acer rubrum*) genotypes compared to water based on LogFC

Rank	Transcript ID	Plants (RPKM)						LogFC	Adj. *p*. value	Description
		Res. 1	Res. 2	Res. 3	Water 1	Water 2	Water 3			
1	TRINITY_DN438303_c0_g1	0.00	0.00	0.00	32.37	51.81	54.69	−10.70	.00208	Purple acid phosphatase
2	TRINITY_DN429524_c0_g2	0.00	0.00	0.00	13.80	29.16	111.83	−9.17	.00666	Putative protein LURP‐one‐related 10‐like
3	TRINITY_DN439956_c0_g1	0.00	0.00	0.00	123.28	50.92	115.24	−8.93	.00208	ACD1‐like
4	TRINITY_DN437970_c0_g1	0.00	0.00	0.58	58.15	27.49	41.18	−8.89	.00649	Putative cyclin B1
5	TRINITY_DN442867_c0_g1	0.00	0.00	0.00	50.14	32.79	8.70	−8.82	.00388	Patatin
6	TRINITY_DN425288_c0_g1	0.00	0.00	0.00	167.32	20.03	213.29	−8.77	.01273	Putative organ‐specific protein S2‐like
7	TRINITY_DN422431_c1_g1	0.00	0.00	0.00	97.09	7.83	180.75	−8.74	.02982	Putative organ‐specific protein P4‐like isoform X2
8	TRINITY_DN431193_c1_g1	0.00	0.00	0.00	166.63	45.63	41.28	−8.73	.00273	Putative 14 kDa proline‐rich protein DC2.15
9	TRINITY_DN433979_c0_g1	0.00	0.00	0.00	9.98	25.74	16.79	−8.62	.00208	AT3 g20370/MQC12_13
10	TRINITY_DN428010_c1_g1	0.00	0.00	0.00	40.35	6.11	8.93	−8.61	.00523	Lipoxygenase
11	TRINITY_DN429852_c0_g1	0.00	0.24	0.66	34.20	75.64	84.42	−8.43	.02260	Sulfotransferase
12	TRINITY_DN435667_c3_g1	0.11	3.94	3.92	591.62	391.57	544.19	−8.41	.01602	Histone H2A
13	TRINITY_DN438161_c2_g3	0.00	0.00	0.08	19.43	18.62	15.06	−8.40	.00208	Putative sphingolipid delta 4 desaturase/C‐4 hydroxylase protein des2
14	TRINITY_DN438742_c0_g1	0.00	0.00	0.00	10.33	10.13	14.72	−8.39	.00208	PHD finger family protein
15	TRINITY_DN433832_c1_g1	0.00	0.00	0.00	136.44	20.03	144.35	−8.33	.00208	Predicted protein
16	TRINITY_DN430711_c1_g1	0.00	0.00	0.00	24.35	34.47	25.46	−8.30	.00208	Pectinesterase
17	TRINITY_DN434996_c3_g3	0.00	0.07	0.00	14.26	8.59	16.56	−8.27	.00212	Coffea canephora DH200 = 94 genomic scaffold
18	TRINITY_DN441538_c0_g2	0.00	0.00	0.00	237.51	326.15	163.70	−8.22	.00208	Putative flavonol synthase/flavanone 3‐hydroxylase
19	TRINITY_DN440736_c1_g2	0.13	0.00	0.00	34.77	9.74	36.29	−8.21	.00307	Expansin B3, BETA 1.6 isoform 1
20	TRINITY_DN442303_c1_g3	0.00	0.00	0.00	18.16	5.98	30.21	−8.20	.00209	Cyclin family protein
21	TRINITY_DN441216_c1_g2	0.00	1.07	1.65	106.58	76.52	112.14	−8.19	.00683	Leucoanthocyanidin reductase
22	TRINITY_DN441930_c4_g8	0.00	0.00	0.00	26.10	14.91	13.68	−8.17	.00208	PREDICTED: epidermis‐specific secreted glycoprotein EP1‐like
23	TRINITY_DN435341_c0_g1	0.00	0.00	2.08	84.71	21.56	42.05	−8.15	.04806	Peroxidase
24	TRINITY_DN432222_c0_g2	0.00	0.48	10.67	160.25	160.56	137.79	−8.15	.00807	4‐coumarate:CoA ligase 3
25	TRINITY_DN441888_c1_g2	0.00	0.00	0.75	31.98	13.24	21.72	−8.06	.00733	Putative alpha‐l‐fucosidase

**Table 3:** Top 25 upregulated transcripts in susceptible red maple (*Acer rubrum*) genotypes compared to water based on LogFC

Rank	Transcript ID	Plants (RPKM)					LogFC	Adj. *p*. Value	Description
		Sus. 1	Sus. 2	Water 1	Water 2	Water 3			
1	TRINITY_DN426651_c2_g1	22.77	344.72	0.00	0.00	0.00	10.43	.04809	Alkaline proteinase
2	TRINITY_DN432097_c0_g1	33.34	25.00	0.00	0.00	0.00	9.98	.00831	Chloride channel protein
3	TRINITY_DN435926_c1_g6	540.37	510.08	0.00	0.00	0.00	9.96	.00831	DCD domain protein isoform 1
4	TRINITY_DN449241_c4_g3	439.64	460.82	0.00	0.00	11.67	9.90	.02447	Putative non‐symbiotic hemoglobin
5	TRINITY_DN446102_c0_g1	376.84	172.26	0.00	0.00	0.00	9.80	.00831	Putative glycine‐rich protein DC7.1
6	TRINITY_DN375151_c0_g1	73.50	25.73	0.00	0.00	0.00	9.79	.00859	60S ribosomal protein L6
7	TRINITY_DN426807_c0_g1	29.20	21.24	0.00	0.00	0.00	9.70	.00831	Albugo candida WGS project CAIX00000000 data
8	TRINITY_DN428352_c1_g1	30.61	31.82	0.00	0.00	1.13	9.70	.02995	Hypothetical protein
9	TRINITY_DN436977_c0_g1	65.96	21.78	0.00	0.00	0.00	9.68	.00881	Cyclin p4
10	TRINITY_DN439029_c0_g7	318.85	353.07	0.00	0.00	0.00	9.67	.00831	NA
11	TRINITY_DN446442_c1_g1	28.25	113.24	0.00	0.00	0.00	9.67	.01058	Non‐symbiotic hemoglobin
12	TRINITY_DN451117_c1_g2	49.99	87.39	0.00	0.00	0.00	9.65	.00831	Putative kinase
13	TRINITY_DN431010_c0_g1	7.49	35.22	0.00	0.00	0.00	9.56	.01218	T1.1 protein
14	TRINITY_DN445195_c1_g1	9.24	9.99	0.00	0.00	0.00	9.50	.00831	Putative cucumisin‐like
15	TRINITY_DN451117_c0_g4	106.48	178.60	0.00	0.00	0.00	9.31	.00831	Kinase‐like protein (Fragment)
16	TRINITY_DN329160_c0_g1	11.83	94.08	0.00	0.00	0.00	9.30	.01858	Putative vegetative cell wall protein gp1‐like
17	TRINITY_DN428750_c1_g1	14.62	65.21	0.00	0.00	0.00	9.24	.00873	Putative tonoplast intrinsic protein
18	TRINITY_DN459004_c0_g1	58.34	36.64	0.00	0.00	0.00	9.18	.00831	RpL31_0 protein (Fragment)
19	TRINITY_DN443554_c2_g4	96.81	1735.86	0.00	0.00	0.00	9.16	.03746	Coffea canephora DH200 = 94 genomic scaffold
20	TRINITY_DN448079_c3_g3	1478.36	695.12	0.00	0.00	0.00	9.15	.00831	Putative TMV resistance protein N‐like
21	TRINITY_DN464912_c0_g1	48.70	26.10	0.00	0.00	0.00	9.15	.00831	TSA: Wollemia nobilis Ref_Wollemi_Transcript_25331_947 transcribed RNA sequence
22	TRINITY_DN427874_c0_g1	221.22	86.41	0.00	2.47	0.00	9.13	.04765	Actophorin
23	TRINITY_DN472589_c0_g1	204.76	59.10	0.95	0.00	0.00	9.13	.04031	C12D12.1 isoform c
24	TRINITY_DN459296_c0_g1	69.67	15.96	0.00	0.00	0.00	9.13	.01085	S9 n
25	TRINITY_DN470902_c0_g1	310.61	68.25	0.00	0.00	0.00	9.11	.01117	Putative 40S ribosomal protein S29

**Table 4:** Top 25 downregulated transcripts in susceptible red maple (*Acer rubrum*) genotypes compared to water based on LogFC

Rank	Transcript ID	Plants (RPKM)					LogFC	Adj. *p* value	Description
		Sus. 1	Sus. 2	Water 1	Water 2	Water 3			
1	TRINITY_DN432222_c0_g2	0.00	0.00	160.25	160.56	137.79	−12.39	.00831	4‐coumarate:CoA ligase 3
2	TRINITY_DN440779_c1_g3	0.00	0.00	325.33	515.61	339.04	−12.30	.00831	Anthocyanidin reductase
3	TRINITY_DN440428_c2_g2	0.00	0.00	87.10	73.13	56.58	−11.82	.00831	Putative AMP dependent CoA ligase
4	TRINITY_DN441216_c1_g2	0.00	0.00	106.58	76.52	112.14	−11.50	.00831	Leucoanthocyanidin reductase
5	TRINITY_DN429852_c0_g1	0.00	0.00	34.20	75.64	84.42	−11.29	.01314	Sulfotransferase
6	TRINITY_DN438303_c0_g1	0.00	0.00	32.37	51.81	54.69	−11.22	.00831	Purple acid phosphatase
7	TRINITY_DN444245_c2_g3	0.00	0.00	55.33	49.80	65.32	−11.18	.00831	Reticulon‐like protein
8	TRINITY_DN447857_c0_g4	0.00	0.00	36.32	10.16	19.42	−10.96	.01573	Cellulose synthase‐like protein D1
9	TRINITY_DN443586_c4_g3	0.00	0.00	440.61	534.18	340.78	−10.89	.00831	Copper transporter
10	TRINITY_DN433607_c0_g1	0.15	0.00	94.48	168.31	69.59	−10.86	.04556	O‐methyltransferase
11	TRINITY_DN430221_c0_g1	0.00	0.00	78.81	68.61	73.84	−10.79	.00831	Limonene synthase
12	TRINITY_DN434133_c0_g3	0.00	0.00	2759.69	7358.98	3725.59	−10.72	.01045	Coffea canephora DH200 = 94 genomic scaffold
13	TRINITY_DN430737_c1_g1	0.00	0.00	49.89	49.63	56.06	−10.64	.00831	Cupredoxin superfamily protein isoform 1
14	TRINITY_DN437199_c0_g1	0.00	0.00	34.95	12.27	23.33	−10.60	.01425	Leucine‐rich repeat‐containing protein 50
15	TRINITY_DN437713_c1_g1	0.00	0.00	58.43	15.11	66.01	−10.58	.03153	Oxidoreductase family
16	TRINITY_DN442519_c0_g1	0.00	0.00	172.08	40.10	55.85	−10.57	.00876	Coffea canephora DH200 = 94 genomic scaffold
17	TRINITY_DN437344_c2_g1	0.00	0.00	375.23	79.16	126.20	−10.56	.02682	TFL1
18	TRINITY_DN445604_c0_g1	0.00	0.00	190.15	58.29	72.74	−10.32	.01208	Putative protein E6
19	TRINITY_DN438732_c0_g1	0.00	0.05	203.84	144.17	140.02	−10.30	.01033	End binding protein 1C isoform 1
20	TRINITY_DN436787_c4_g2	0.00	0.00	27.74	22.67	34.88	−10.28	.00831	Fatty acid/sphingolipid desaturase
21	TRINITY_DN436541_c0_g1	0.00	0.00	172.83	12.78	31.52	−10.25	.02255	Sulfotransferase
22	TRINITY_DN430929_c3_g1	0.00	0.00	39.49	15.22	82.23	−10.23	.02661	Putative benzoate carboxyl methyltransferase
23	TRINITY_DN449002_c2_g1	0.00	0.00	14.54	17.02	14.11	−10.03	.00831	Leucine‐rich repeat protein kinase family protein isoform 1
24	TRINITY_DN433129_c0_g3	0.00	0.00	19.65	51.10	9.66	−9.97	.00831	DUF594 family protein
25	TRINITY_DN441888_c1_g2	0.00	0.00	31.98	13.24	21.72	−9.87	.00975	Putative alpha‐l‐fucosidase

**Figure 4:**
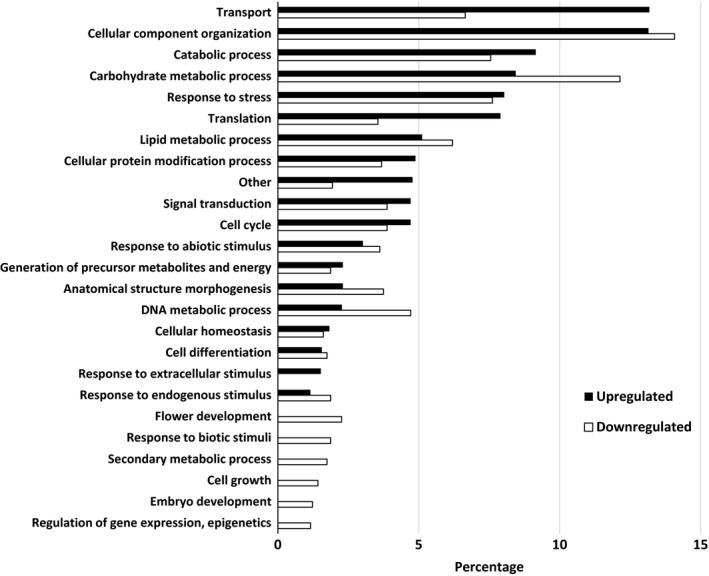
Percentage of differentially expressed upregulated and downregulated transcripts when nickel resistant red maple (*Acer rubrum*) genotypes were compared to water controls. For upregulated and downregulated transcripts, 2,951 and 1,549 transcripts were identified and classified by biological function based on their gene ontology term using the BLAST2GO software

**Figure 5:**
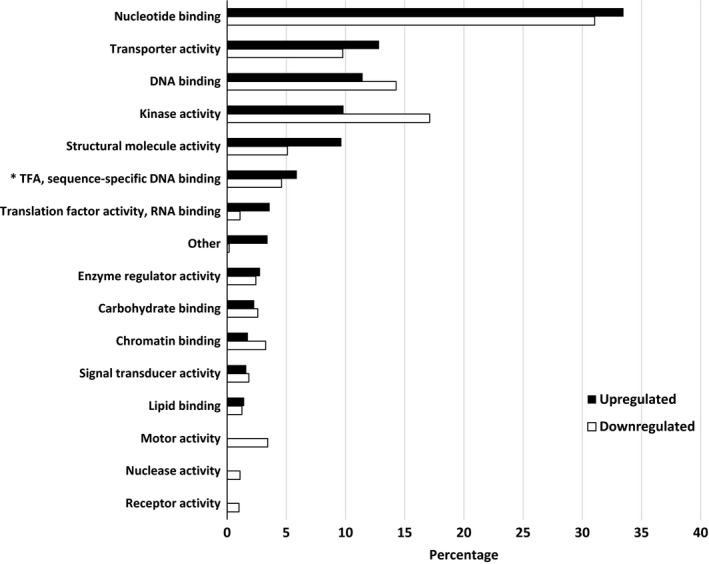
Percentage of differentially expressed upregulated and downregulated transcripts when nickel resistant red maple (*Acer rubrum*) genotypes were compared to water controls. For upregulated and downregulated transcripts, 2,228 and 1,198 were identified and classified by molecular function based on their gene ontology term using the BLAST2GO software. *TFA stands for Transcription factor activity

**Figure 6:**
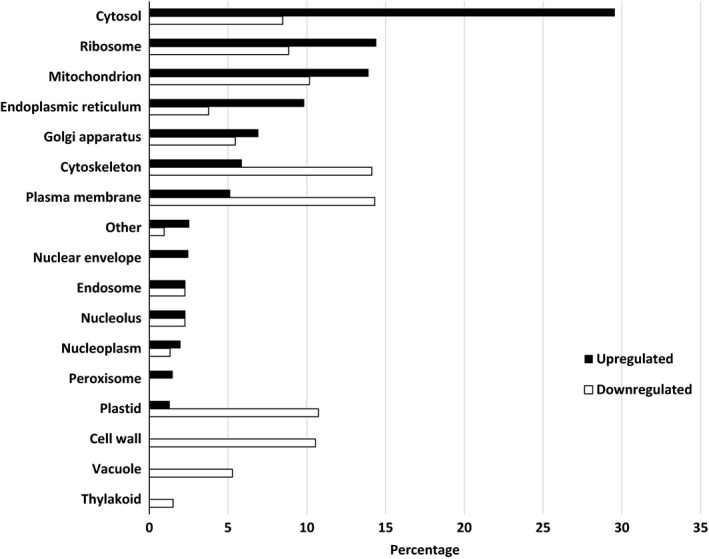
Percentage of differentially expressed upregulated and downregulated transcripts when nickel resistant red maple (*Acer rubrum*) genotypes were compared to water controls. For upregulated and downregulated transcripts, 1,617 and 531 were identified and classified by cellular compartment based on their gene ontology term using the BLAST2GO software

Pairwise comparisons for biological process, molecular functions, and cellular compartments between RG and water and SG and water are described in Figures 4, 5, 6. For biological process, the most differentially expressed transcripts coded for proteins associated with transport, cellular component organization, catabolic process, carbohydrate metabolic process, response to stress, translation, lipid metabolic process, and cellular modification process when RG were compared to water (Figure 4). There was twice the number of upregulated compared to downregulated transcripts that coded for transporters and proteins involved in translation activities in RG compared to water control. A small proportion of differentially expressed transcripts translated proteins associated with flower development, response to biotic stimuli, secondary metabolic process, cell growth, embryo development, and regulation of gene expression and epigenetics. For molecular function, over 30% of the transcripts that were upregulated or downregulated coded for proteins that were associated with nucleotide binding (Figure 5). There was more downregulated (17%) than upregulated (9.5%) transcripts coding for proteins associated with kinase activity when RG were compared to controls. For cellular component, most differentially expressed transcripts coded for proteins found in the cytosol. The majority of these transcripts were upregulated as there were three times more upregulated than downregulated transcripts in that category. A different trend was observed in the cytoskeleton, plasma membrane, plastid, with more downregulated than upregulated. The transcripts coding for proteins found in the cell wall, vacuole, and thylakoid were all downregulated in RG when compared to water (Figure 6). Hence, upregulation of genes associated with transport in cytosol is the main mechanism involved in RG in the presence of Ni.

**Figure 7:**
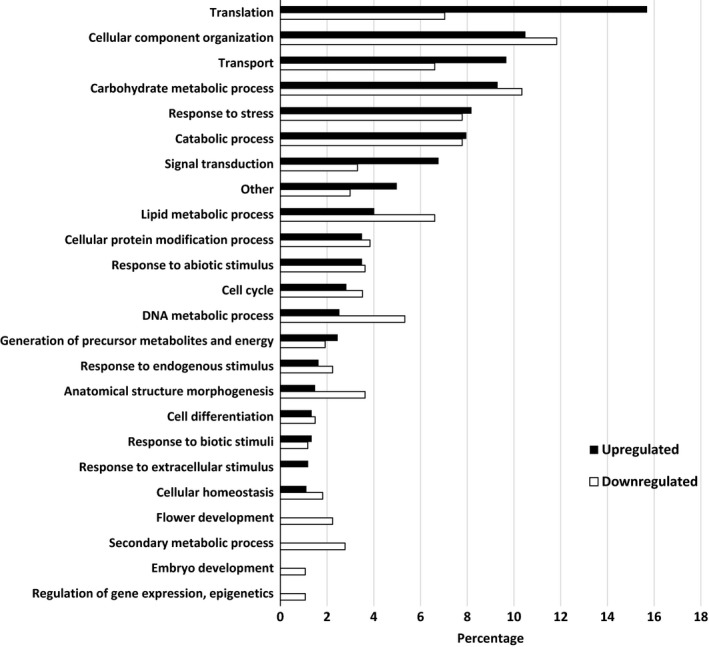
Percentage of differentially expressed upregulated and downregulated transcripts when nickel susceptible red maple (*Acer rubrum*) genotypes were compared to water controls. For upregulated and downregulated transcripts, 1,344 and 938 were identified and classified by biological function based on their gene ontology (GO) term using the BLAST2GO software

**Figure 8:**
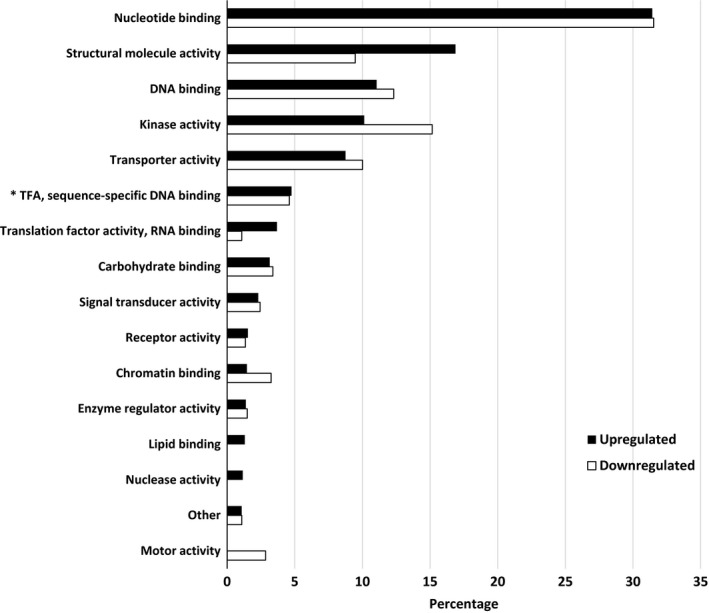
Percentage of differentially expressed upregulated and downregulated transcripts when nickel susceptible red maple (*Acer rubrum*) genotypes were compared to water controls. For upregulated and downregulated transcripts, 1,303 and 739 were identified and classified by molecular function based on their gene ontology term using the BLAST2GO software. *TFA stands for transcription factor activity

**Figure 9:**
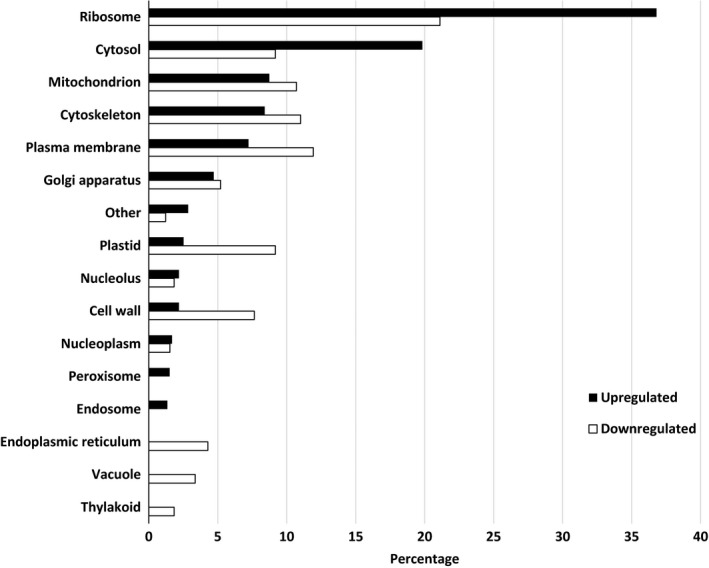
Percentage of differentially expressed upregulated and downregulated transcripts when nickel susceptible red maple (*Acer rubrum*) genotypes were compared to water controls. For upregulated and downregulated transcripts, 595 and 327 were identified and classified by cellular compartment based on their gene ontology term using the BLAST2GO software

When SG were compared to water, the top biological processes were translation, cellular component organization, transport, carbohydrate metabolic process, response to stress, catabolic process, and signal transduction (Figure 7). There were more upregulated transcripts than downregulated for translation and signal transduction while no difference between upregulation and downregulation was observed for genes associated with the other main biological processes. A downregulation was observed for transcripts coding for proteins associated with lipid metabolic processes and anatomical structure morphogenesis. As with RG, few downregulated transcripts coded for proteins associated with flower development, secondary metabolic process, embryo development, and regulation of gene expression and epigenetics. For molecular function, the pattern of gene regulation was similar to that observed when RG were compared to water (Figure 8). Transcripts coding for proteins associated with nucleotide binding were the most prevalent with equal amount of upregulation and downregulation. For cellular component, most differentially expressed transcripts coded for proteins in the ribosome. These transcripts were more upregulated than downregulated. The same trend was observed for the transcripts in the cytosol. On the other hand, there was a higher downregulation than upregulation in the plastid and cell wall. The few differentially expressed transcripts that coded for proteins found in the endoplasmic reticulum, vacuole, and thylakoid were downregulated (Figure 9). Overall, upregulation of genes in ribosome is the dominant mechanism involved in SG in the presence of Ni.

## 4 DISCUSSION

### 4.1 Genetic resistance to nickel

Because of their lack of mobility, plants are continuously exposed to abiotic stresses. They have developed an array of morphological, physiological, and biochemical responses that allow them to tolerate or avoid these stressors. In the present study, *A. rubrum*, a Ni avoider, showed a high level of resistance to nickel toxicity as no damage was observed when treated with a high dose of nickel at 1,600 mg/kg dry soil. Only three plants showed delayed expression of nickel susceptibility and were used for gene regulation analysis. A recent study revealed that silver maple (*A. saccharinum*), a closely related species to *A. rubrum,* is also highly resistant to Ni suggesting that this resistance might be a common characteristic of the *Acer* genus. But, the two species use different physiological mechanisms to deal with Ni contamination, *A. sacharinum* being a Ni excluder and *A. rubrum*, a Ni avoider. (Nkongolo et al., 2017).

### 4.2 Gene expression and ontology analyses

We found no difference in gene expression at high stringency (FDR >0.05) between Ni resistant and susceptible genotypes. The heatmap clustering also showed that the two types of genotype (RG and SG) form a distinct cluster that was separated from the control groups. RG and SG samples intermingled within the same cluster. Some differences in gene expression were observed at low stringency based on a *p*‐value analysis. But, *p*‐value is more susceptible to including false‐positive results than the False Discovery Rate. Hence, the results based on the *p*‐values analysis were not considered in the analysis of gene expression. Significant differences between RG and SG were found when they were compared to water. The validation of DEG results by qPCR was not necessary because a series of quality controls during assembly and sequencing, and data processing was performed. In addition, data were analyzed using stringent statistical tests. Moreover, we used two types of references (water and nitrate controls) to filter the effect of nitrate on gene expression. Furthermore, the amount of RNA recovered specially in susceptible genotypes was not enough to run other types of validation analysis such as qPCR.

In general, gene ontology (GO) revealed relevant information on possible mechanisms involved in Ni resistance in *A. rubrum*. GO defines gene functions and how these functions are related to each other. It describes molecular function (molecular‐level activities performed by gene products), biological process (the larger processes, or ‘biological programs), and cellular components (the locations relative to cellular structures in which a gene product performs a function) (Gene Ontology Consortium, 2017). In the present study, there was an upregulation of genes associated with transport in cytosol in RG compared to water control while upregulation of genes associated with translation in the ribosome was prevalent in susceptible genotypes when compared to the same control. In contrast, Theriault et al. (2016b) reported that the main mechanism involved in nickel resistance in *B. papyrifera,* a Ni accumulator, is a downregulation of genes associated with translation (in ribosome), binding, and transporter activities.

Moreover, in *B. papyrifera*, six candidate genes associated with nickel resistance were identified (Theriault et al., 2016b). They include Glutathione S–transferase, thioredoxin family protein, putative transmembrane protein, Nramp transporter, TonB receptor, and TonB‐dependant protein. Detailed analysis of the *A. rubrum* transcriptome revealed no specific gene that could be associated with nickel resistance. Unlike *B. papyrifera*, the results of the present study suggest that there are no major genes that could be associated with Ni resistance in *A. rubrum*. The majority of the DEG between resistant and control are the same as between susceptible and control. The small differences between RG and SG were not detected at the high level of stringency (False Discovery Rate) used in the statistical analysis. They were unrevealed when comparing both types of genotypes (RG and SG) to water. Alkaline proteinase (AP) was highly upregulated in both RG and SG compared to water. This extreme level of upregulation of AP triggered by nickel treatment has not been reported in any other organisms.

Overall, the complexity of the mechanism involved in nickel resistance in *A. rubrum* could be associated with the physiological process used by this species to cope with nickel contamination. The metal avoidance in plants such as *A. rubrum* is associated with morphological changes at the root system and likely involves auxins (Cai et al., 2011; Khare et al., 2017; Liu et al., 2011; Overvoorde, Fukaki, & Beeckman, 2010; Potters et al., 2007; Vitti et al., 2014). Plants also limit metal assimilation by the roots by secreting a number of substances such as organic acids, and substances in root extracellular matrix such as sugars, phenols, amino acids, and polysaccharides (Cai et al., 2011; Guo, Liang, & Zhu, 2009; Jutsz & Gnida, 2015). The mechanism used by *A. rubrum* to avoid nickel is unclear, but considering that the nickel treatment was conducted in a controlled environment using a sterilized sand / soil mix, it is likely that the avoidance mechanism is *in situ*. Khare et al. (2017) demonstrated that root avoidance of toxic metals requires GLABRA1 Enhancer‐Binding Protein (GeBP) transcription factor (TF; GPL4) in *Arabidopsis thaliana*. Such mechanism has not yet been demonstrated in other plant species or with metals other than cadmium.

Review of existing literature shows that reports on the mechanisms of metal resistance have focussed on six main areas (a) uptake kinetics, (b) metabolism, (c) complexation, (d) redox stress, (e) subcellular localization, and (f) intracellular localization (Meharg, 2005). Subcellular and intracellular localization are unlikely in *A. rubrum* considering the absence of Ni accumulation in its tissues. The lack of expression of glutathione associated genes that play a key role in redox stress processes suggests that this mechanism is not involved as well in *A. rubrum* response to nickel contamination (Hartley‐Whitaker, Ainsworth, & Meharg, 2001; Hartley‐Whitaker, Woods, & Meharg, 2002; Meharg, 2005). Our data suggest that uptake kinetics and metabolisms are the key processes involved in *A. rubrum* reaction to Ni toxicity. Mechanisms for protecting plants from oxidative stress appear to be constitutive in both resistant and sensitive genotypes.

The results of the present study suggest that resistance genes preexist in the genome of *A. rubrum* considering that the seeds used for this investigation are from an *A. rubrum* population that was not previously exposed to Ni. Other studies have reported Ni resistance in genotypes from metal‐contaminated sites (Kirkey, Matthews, & Ryser, 2012; Watmough & Hutchinson, 1997). The fact that no major genes associated with metal transport or processing were identified in the analysis of the RG and SG *A. rubrum* transcriptome suggests that the genetic mechanism controlling the response of this species to nickel is controlled by several genes with limited expression. The subtle differences between resistant and susceptible in gene regulation, if they exist, were difficult to detect in direct pairwise comparison.

## 5 CONCLUSION

The main goal of the present study was to determine the mechanism involved in *A. rubrum* response to nickel toxicity. Nickel treatment at a high dose of 1,600 mg/kg triggers regulation of several genes. The study revealed that unlike *B. papyrifera*, there were no significant differences in genes expression when RG and SG were compared based on the FDR test. However, distinctive differences between the two groups were found when they were compared to water controls. Upregulation of genes associated with transport in cytosol is the main mechanism associated with response to Ni in RG while upregulation of genes involved in translation in the ribosome was prevalent in susceptible genotypes in comparison with control. No major genes associated with nickel resistance were identified. Several highly expressed genes were found in the top 100 upregulated and downregulated based on heatmap profiles and logFC analysis. This suggests that the genetic mechanism controlling the resistance of *A. rubrum* to nickel is controlled by genes with limited effects.

## AVAILABILITY OF DATA AND MATERIALS

We have deposited data in the following depository:

Repository/DataBank Accession: EmbL
SRA project number: SRP098922.
Databank URL: http://www.ebi.ac.uk/ena

Repository/DataBank Accession: NCBI gene bank
SRA project number: SRP098922.
Databank URL: http://www.ncbi.nlm.nih.gov/genbank

## ETHICS APPROVAL AND CONSENT TO PARTICIPATE

Not applicable.

## CONSENT TO PUBLISH

Not applicable.

## CONFLICT OF INTEREST

The authors declare that they have no conflict of interest.

## AUTHOR CONTRIBUTION

KNK designed the research, and monitored the experiments, and wrote the manuscript; GT performed the experiments, analyzed the data, and reviewed the manuscript; PM supervised the experiments and reviewed the data and the manuscript.
