# Supplementary material for: Differential levels of gene expression and molecular mechanisms between red maple (Acer rubrum) genotypes resistant and susceptible to nickel toxicity revealed by transcriptome analysis
Source: Ecol Evol. 2018 Apr 19;8(10):4876–90. doi: 10.1002/ece3.4045 (PMC5980433; doi:10.1002/ece3.4045)
Supplement: Supplementary file 1 [file ECE3-8-4876-s001.docx]

**Supplementary materials**

**Table 1S. Top 100 (LogFC) upregulated transcripts in resistant red maple (*Acer rubrum*) genotypes compared to water controls.**

| **Rank** | **Transcript ID** | **Plants (RPKM)** | | | | | | **LogFC** | **Adj. P.Value** | **Description** |
| --- | --- | --- | --- | --- | --- | --- | --- | --- | --- | --- |
|  |  | **Res. 1** | **Res. 2** | **Res. 3** | **H_2_O 1** | **H_2_O 2** | **H_2_O 3** |  |  |  |
| 1 | TRINITY_DN426651_c2_g1 | 159.05 | 366.63 | 365.78 | 0.00 | 0.00 | 0.00 | 11.99 | 0.00208 | Alkaline proteinase |
| 2 | TRINITY_DN420778_c2_g4 | 78.36 | 142.49 | 169.90 | 0.00 | 0.00 | 0.04 | 11.87 | 0.00209 | Proteinase T |
| 3 | TRINITY_DN425833_c0_g1 | 26.79 | 58.46 | 56.29 | 0.00 | 0.00 | 0.00 | 11.64 | 0.00208 | Predicted protein |
| 4 | TRINITY_DN428667_c1_g1 | 64.11 | 146.03 | 68.19 | 0.00 | 0.00 | 0.00 | 11.54 | 0.00208 | GRF domain class transcription factor |
| 5 | TRINITY_DN437630_c5_g2 | 75.50 | 329.35 | 204.28 | 0.00 | 0.00 | 0.00 | 10.81 | 0.00228 | Ribosomal protein S30 |
| 6 | TRINITY_DN425283_c1_g1 | 538.38 | 925.45 | 985.42 | 0.00 | 0.00 | 18.17 | 10.80 | 0.01728 | CFEM-domain-containing protein |
| 7 | TRINITY_DN422756_c0_g1 | 154.07 | 587.86 | 398.80 | 0.00 | 0.00 | 0.00 | 10.75 | 0.00208 | Elongation factor 1-alpha |
| 8 | TRINITY_DN402821_c0_g1 | 17.72 | 45.29 | 22.73 | 0.00 | 0.00 | 0.00 | 10.71 | 0.00208 | Glycoside hydrolase family 54 protein |
| 9 | TRINITY_DN431004_c1_g4 | 83.01 | 492.12 | 298.61 | 0.00 | 0.00 | 0.00 | 10.70 | 0.00327 | Ribosomal protein |
| 10 | TRINITY_DN428052_c1_g1 | 43.29 | 244.97 | 173.21 | 0.00 | 0.00 | 0.41 | 10.60 | 0.04443 | Kelch repeat protein |
| 11 | TRINITY_DN373295_c0_g2 | 61.42 | 326.93 | 157.24 | 0.00 | 0.00 | 0.00 | 10.46 | 0.00254 | Predicted protein |
| 12 | TRINITY_DN432608_c1_g1 | 53.48 | 249.63 | 177.29 | 0.00 | 0.00 | 0.37 | 10.45 | 0.02171 | CFEM-domain-containing protein |
| 13 | TRINITY_DN426185_c1_g2 | 11.53 | 78.81 | 45.91 | 0.00 | 0.00 | 0.00 | 10.43 | 0.00362 | GPI anchored serine-rich protein |
| 14 | TRINITY_DN389300_c0_g2 | 21.12 | 171.08 | 39.68 | 0.00 | 0.00 | 0.00 | 10.31 | 0.00418 | Endo-1,4-beta-glucanase |
| 15 | TRINITY_DN436844_c0_g1 | 23.67 | 160.07 | 48.99 | 0.00 | 0.00 | 0.00 | 10.30 | 0.00315 | Predicted protein |
| 16 | TRINITY_DN435092_c1_g1 | 14.67 | 94.06 | 20.07 | 0.00 | 0.00 | 0.09 | 10.28 | 0.02697 | WGS project CABT00000000 data |
| 17 | TRINITY_DN432457_c1_g3 | 18.66 | 90.77 | 61.94 | 0.00 | 0.00 | 0.00 | 10.26 | 0.00327 | Putative ubiquitin conjugating enzyme (UbcD) |
| 18 | TRINITY_DN418791_c0_g1 | 49.13 | 203.79 | 60.55 | 0.00 | 0.00 | 0.00 | 10.19 | 0.00210 | Predicted protein |
| 19 | TRINITY_DN426651_c1_g1 | 57.06 | 143.20 | 136.41 | 0.00 | 0.00 | 0.00 | 10.18 | 0.00208 | Alkaline proteinase |
| 20 | TRINITY_DN436706_c1_g2 | 18.57 | 85.44 | 39.26 | 0.00 | 0.00 | 0.00 | 10.17 | 0.00209 | Probable ribosomal protein L9.e.c14 |
| 21 | TRINITY_DN405758_c1_g1 | 7.43 | 104.65 | 19.32 | 0.00 | 0.00 | 0.00 | 10.17 | 0.00775 | Mannanase |
| 22 | TRINITY_DN439596_c1_g1 | 106.96 | 36.90 | 36.58 | 0.00 | 0.00 | 0.00 | 10.15 | 0.00208 | Putative growth factor independence |
| 23 | TRINITY_DN424010_c0_g2 | 4.85 | 34.24 | 23.09 | 0.00 | 0.00 | 0.00 | 10.14 | 0.00368 | Prenylated Rab acceptor 1 |
| 24 | TRINITY_DN431808_c3_g3 | 46.13 | 206.90 | 115.58 | 0.00 | 0.00 | 0.00 | 10.14 | 0.00210 | Chromosome 4, complete genome |
| 25 | TRINITY_DN447378_c2_g1 | 40.06 | 27.74 | 79.39 | 0.00 | 0.00 | 0.00 | 10.13 | 0.00209 | Glycosyltransferase |
| 26 | TRINITY_DN425980_c1_g2 | 5.68 | 45.97 | 25.57 | 0.00 | 0.00 | 0.00 | 10.12 | 0.00388 | Cell wall protein |
| 27 | TRINITY_DN423641_c2_g3 | 5.50 | 16.11 | 9.34 | 0.00 | 0.00 | 0.00 | 10.08 | 0.00208 | 1,3-beta-glucan synthase component bgs3 |
| 28 | TRINITY_DN450340_c1_g4 | 465.95 | 131.89 | 217.01 | 0.00 | 0.00 | 0.00 | 10.05 | 0.00208 | CYP81Q32 |
| 29 | TRINITY_DN427051_c3_g1 | 3.78 | 20.11 | 16.19 | 0.00 | 0.00 | 0.00 | 10.04 | 0.00276 | Alpha-1,4 glucan phosphorylase CBS 393.64 |
| 30 | TRINITY_DN425996_c0_g1 | 205.44 | 635.89 | 335.12 | 0.00 | 0.00 | 4.19 | 10.04 | 0.01971 | Hypothetical protein AOR_1_1366184 |
| 31 | TRINITY_DN409623_c1_g1 | 20.80 | 302.07 | 63.17 | 0.00 | 0.00 | 0.15 | 10.02 | 0.03744 | Glycosyl hydrolase family 61 |
| 32 | TRINITY_DN449832_c0_g1 | 11.29 | 55.98 | 18.60 | 0.00 | 0.00 | 0.00 | 9.99 | 0.00225 | Glycoside hydrolase family 93 |
| 33 | TRINITY_DN440752_c0_g1 | 14.26 | 77.43 | 61.48 | 0.00 | 0.00 | 0.00 | 9.99 | 0.00294 | Methyltransferase domain-containing protein |
| 34 | TRINITY_DN418601_c0_g2 | 6.99 | 24.41 | 30.96 | 0.00 | 0.00 | 0.00 | 9.98 | 0.00215 | Predicted protein |
| 35 | TRINITY_DN440872_c0_g1 | 257.11 | 625.43 | 543.44 | 0.00 | 0.00 | 0.00 | 9.98 | 0.00208 | Trypsin-like protease n=4 |
| 36 | TRINITY_DN441929_c1_g4 | 25.11 | 27.63 | 70.15 | 0.00 | 0.00 | 0.00 | 9.95 | 0.00208 | Putative F-box protein At3g25750 |
| 37 | TRINITY_DN420361_c0_g1 | 5.02 | 48.40 | 27.38 | 0.00 | 0.00 | 0.00 | 9.90 | 0.00481 | Glycoside hydrolase family 64 protein |
| 38 | TRINITY_DN432999_c0_g3 | 50.50 | 207.33 | 115.90 | 0.00 | 0.00 | 1.15 | 9.90 | 0.03071 | Woronin body major protein-like protein |
| 39 | TRINITY_DN433026_c1_g1 | 4.64 | 40.76 | 43.56 | 0.00 | 0.00 | 0.00 | 9.89 | 0.00549 | Predicted protein |
| 40 | TRINITY_DN463178_c0_g1 | 17.70 | 55.74 | 32.13 | 0.00 | 0.00 | 0.00 | 9.88 | 0.00208 | Cutinase |
| 41 | TRINITY_DN433554_c0_g1 | 115.37 | 155.51 | 81.90 | 0.00 | 0.00 | 0.00 | 9.88 | 0.00208 | 17.5 kd heat shock family protein |
| 42 | TRINITY_DN421391_c0_g1 | 9.22 | 94.00 | 19.33 | 0.00 | 0.00 | 0.00 | 9.86 | 0.00475 | Alpha-L-arabinofuranosidase |
| 43 | TRINITY_DN396774_c0_g1 | 8.27 | 25.71 | 12.70 | 0.00 | 0.00 | 0.00 | 9.86 | 0.00208 | Glycoside hydrolase family 2 |
| 44 | TRINITY_DN418800_c1_g1 | 3.52 | 41.28 | 26.00 | 0.00 | 0.00 | 0.00 | 9.85 | 0.00667 | Predicted protein n=1 |
| 45 | TRINITY_DN419762_c0_g1 | 5.42 | 35.88 | 9.73 | 0.00 | 0.00 | 0.00 | 9.85 | 0.00293 | Beta-glucosidase |
| 46 | TRINITY_DN429076_c4_g2 | 7.46 | 81.20 | 27.38 | 0.00 | 0.00 | 0.04 | 9.83 | 0.01328 | Glycosylhydrolase family 7-1 |
| 47 | TRINITY_DN422221_c0_g1 | 3.97 | 68.03 | 37.23 | 0.00 | 0.00 | 0.00 | 9.80 | 0.01042 | Predicted protein |
| 48 | TRINITY_DN394590_c0_g2 | 5.39 | 62.29 | 9.22 | 0.00 | 0.00 | 0.00 | 9.79 | 0.00583 | Endo-1,4-beta-glucanase |
| 49 | TRINITY_DN414169_c0_g1 | 4.29 | 28.95 | 32.75 | 0.00 | 0.00 | 0.00 | 9.78 | 0.00426 | Mutanase |
| 50 | TRINITY_DN434934_c0_g2 | 40.82 | 227.70 | 111.22 | 0.00 | 0.00 | 0.00 | 9.77 | 0.00230 | Ribosomal protein S29, S14 family |
| 51 | TRINITY_DN422594_c0_g1 | 8.30 | 43.48 | 36.61 | 0.00 | 0.00 | 0.00 | 9.77 | 0.00264 | Predicted protein |
| 52 | TRINITY_DN432815_c4_g2 | 23.16 | 241.05 | 161.13 | 0.00 | 0.00 | 0.00 | 9.76 | 0.00555 | Elongation factor 1-alpha |
| 53 | TRINITY_DN432905_c4_g3 | 10.20 | 32.72 | 44.11 | 0.00 | 0.00 | 0.00 | 9.75 | 0.00208 | Transaldolase |
| 54 | TRINITY_DN430296_c0_g1 | 9.04 | 41.12 | 22.45 | 0.00 | 0.00 | 0.00 | 9.74 | 0.00209 | Predicted protein |
| 55 | TRINITY_DN435263_c4_g1 | 50.41 | 209.41 | 118.66 | 0.00 | 0.00 | 0.00 | 9.72 | 0.00208 | 40S ribosomal protein S23 |
| 56 | TRINITY_DN431010_c0_g1 | 18.81 | 61.97 | 6.07 | 0.00 | 0.00 | 0.00 | 9.71 | 0.00429 | T1.1 protein |
| 57 | TRINITY_DN451017_c3_g7 | 2027.67 | 3435.56 | 1280.87 | 0.00 | 66.17 | 0.00 | 9.70 | 0.00683 | NAD(P)-linked oxidoreductase superfamily protein |
| 58 | TRINITY_DN43011_c0_g1 | 39.15 | 142.04 | 30.34 | 0.00 | 0.00 | 0.79 | 9.70 | 0.04612 | Alpha-L-arabinofuranosidase |
| 59 | TRINITY_DN437425_c6_g3 | 20.20 | 106.00 | 104.88 | 0.12 | 0.00 | 0.00 | 9.69 | 0.00529 | Peptidyl-prolyl cis-trans isomerase |
| 60 | TRINITY_DN440532_c2_g1 | 20.43 | 67.66 | 38.78 | 0.00 | 0.00 | 0.00 | 9.67 | 0.00208 | Guanine nucleotide-binding protein subunit beta-like protein |
| 61 | TRINITY_DN433729_c2_g3 | 8.79 | 34.25 | 15.52 | 0.00 | 0.00 | 0.00 | 9.65 | 0.00208 | Carboxypeptidase A |
| 62 | TRINITY_DN427859_c5_g3 | 37.88 | 313.22 | 85.20 | 0.00 | 0.00 | 0.28 | 9.63 | 0.01355 | Predicted protein |
| 63 | TRINITY_DN429531_c3_g5 | 1.69 | 14.33 | 9.57 | 0.00 | 0.00 | 0.00 | 9.60 | 0.00408 | Predicted protein |
| 64 | TRINITY_DN417293_c1_g1 | 8.79 | 27.49 | 19.62 | 0.00 | 0.00 | 0.00 | 9.57 | 0.00208 | Peptide hydrolase |
| 65 | TRINITY_DN415715_c0_g2 | 2.29 | 13.25 | 14.98 | 0.00 | 0.00 | 0.00 | 9.55 | 0.00336 | Cation transporter-like protein |
| 66 | TRINITY_DN423546_c0_g3 | 3.60 | 31.92 | 17.40 | 0.00 | 0.00 | 0.00 | 9.54 | 0.00290 | Fusarium graminearum chromosome 2, complete genome |
| 67 | TRINITY_DN421546_c0_g1 | 4.27 | 23.86 | 39.84 | 0.00 | 0.00 | 0.00 | 9.53 | 0.00426 | Predicted protein |
| 68 | TRINITY_DN433647_c2_g1 | 3.19 | 25.36 | 12.25 | 0.00 | 0.00 | 0.00 | 9.52 | 0.00336 | Glycoside hydrolase family 17 protein (Fragment) |
| 69 | TRINITY_DN433234_c4_g8 | 101.16 | 408.77 | 137.68 | 0.00 | 0.00 | 0.00 | 9.51 | 0.00209 | Putative 60S ribosomal protein L8 |
| 70 | TRINITY_DN429713_c4_g1 | 5.28 | 92.65 | 26.49 | 0.00 | 0.00 | 0.00 | 9.51 | 0.00282 | Fructose bisphosphate aldolase |
| 71 | TRINITY_DN424604_c0_g1 | 3.55 | 41.80 | 31.31 | 0.00 | 0.00 | 0.00 | 9.50 | 0.00651 | Predicted protein |
| 72 | TRINITY_DN425242_c0_g1 | 4.92 | 14.26 | 24.66 | 0.00 | 0.00 | 0.00 | 9.50 | 0.00210 | Predicted protein |
| 73 | TRINITY_DN439689_c8_g2 | 7.99 | 50.95 | 32.72 | 0.00 | 0.00 | 0.00 | 9.50 | 0.00232 | Elongation factor 2 |
| 74 | TRINITY_DN407838_c0_g1 | 10.62 | 21.64 | 16.85 | 0.00 | 0.00 | 0.00 | 9.49 | 0.00208 | Zinc carboxypeptidase |
| 75 | TRINITY_DN425700_c1_g1 | 4.88 | 22.85 | 19.29 | 0.00 | 0.00 | 0.00 | 9.48 | 0.00226 | Glycerol kinase-like protein |
| 76 | TRINITY_DN431192_c3_g2 | 13.41 | 47.29 | 34.48 | 0.00 | 0.00 | 0.00 | 9.48 | 0.00208 | Ribosomal protein L28 |
| 77 | TRINITY_DN440872_c1_g1 | 56.04 | 192.51 | 129.24 | 0.00 | 0.00 | 0.97 | 9.48 | 0.01059 | Serin endopeptidase |
| 78 | TRINITY_DN438386_c0_g1 | 8.46 | 42.97 | 37.82 | 0.00 | 0.00 | 0.00 | 9.47 | 0.00299 | Inorganic pyrophosphatase |
| 79 | TRINITY_DN411266_c0_g1 | 7.22 | 39.06 | 6.08 | 0.00 | 0.00 | 0.04 | 9.46 | 0.01133 | Beta-galactosidase |
| 80 | TRINITY_DN408982_c0_g2 | 7.64 | 15.77 | 17.06 | 0.00 | 0.00 | 0.00 | 9.44 | 0.00208 | Putative fad fmn-containing dehydrogenase protein |
| 81 | TRINITY_DN449710_c1_g1 | 4.29 | 48.19 | 24.17 | 0.00 | 0.00 | 0.00 | 9.43 | 0.00367 | Elongation factor 2 |
| 82 | TRINITY_DN442555_c0_g1 | 87.10 | 37.67 | 22.44 | 0.00 | 0.08 | 0.00 | 9.40 | 0.00208 | Putative inactive poly [ADP-ribose] polymerase SRO2 |
| 83 | TRINITY_DN395711_c0_g1 | 6.55 | 42.70 | 37.58 | 0.00 | 0.00 | 0.00 | 9.40 | 0.00325 | Superoxide dismutase |
| 84 | TRINITY_DN429314_c0_g4 | 4.11 | 37.86 | 18.73 | 0.00 | 0.00 | 0.00 | 9.40 | 0.00367 | Glycoside hydrolase family 16 protein |
| 85 | TRINITY_DN422735_c0_g1 | 15.60 | 52.64 | 29.32 | 0.00 | 0.00 | 0.00 | 9.39 | 0.00208 | Ribosomal L28e family protein |
| 86 | TRINITY_DN408151_c0_g1 | 11.12 | 59.91 | 33.51 | 0.00 | 0.00 | 0.07 | 9.39 | 0.01717 | Ergosterol biosynthesis ERG4/ERG24 family protein |
| 87 | TRINITY_DN329659_c0_g1 | 8.23 | 100.83 | 35.43 | 0.00 | 0.00 | 0.06 | 9.38 | 0.01323 | Predicted protein |
| 88 | TRINITY_DN413617_c0_g1 | 32.81 | 18.43 | 13.07 | 0.00 | 0.00 | 0.00 | 9.38 | 0.00208 | Alcohol oxidase |
| 89 | TRINITY_DN391533_c0_g2 | 13.28 | 55.97 | 49.92 | 0.00 | 0.00 | 0.07 | 9.38 | 0.00374 | Epl1 protein |
| 90 | TRINITY_DN417245_c0_g3 | 9.65 | 53.63 | 19.67 | 0.00 | 0.00 | 0.00 | 9.37 | 0.00228 | Predicted protein |
| 91 | TRINITY_DN438144_c0_g1 | 191.76 | 3448.72 | 99.03 | 0.00 | 0.00 | 0.78 | 9.36 | 0.02234 | Putative transmembrane protein |
| 92 | TRINITY_DN440132_c0_g2 | 6.23 | 17.40 | 10.67 | 0.00 | 0.00 | 0.00 | 9.34 | 0.00208 | Strain CBS 89968 unplaced genomic scaffold supercont1.4, whole genome shotgun sequence |
| 93 | TRINITY_DN437488_c3_g1 | 15.14 | 101.10 | 33.32 | 0.00 | 0.00 | 0.00 | 9.34 | 0.00254 | Fusarium graminearum chromosome 3, complete genome |
| 94 | TRINITY_DN379582_c0_g1 | 1.94 | 10.65 | 7.51 | 0.00 | 0.00 | 0.00 | 9.33 | 0.00241 | Calcium-transporting ATPase |
| 95 | TRINITY_DN432373_c0_g1 | 15.87 | 77.75 | 42.76 | 0.00 | 0.00 | 0.96 | 9.33 | 0.04775 | Putative alcohol oxidase protein |
| 96 | TRINITY_DN376726_c0_g1 | 17.73 | 68.44 | 50.95 | 0.00 | 0.00 | 0.00 | 9.31 | 0.00208 | Peptidyl-prolyl cis-trans isomerase |
| 97 | TRINITY_DN432710_c0_g1 | 11.23 | 60.92 | 36.17 | 0.00 | 0.00 | 0.10 | 9.29 | 0.00672 | Predicted protein |
| 98 | TRINITY_DN406305_c0_g1 | 21.12 | 36.63 | 28.21 | 0.00 | 0.00 | 0.00 | 9.27 | 0.00208 | Predicted protein |
| 99 | TRINITY_DN418134_c3_g1 | 37.29 | 176.83 | 101.97 | 0.00 | 0.00 | 0.68 | 9.27 | 0.01263 | Predicted protein |
| 100 | TRINITY_DN428104_c0_g2 | 3.91 | 11.49 | 11.03 | 0.00 | 0.00 | 0.00 | 9.27 | 0.00208 | Fusarium graminearum chromosome 2, complete genome |

**Table 2S. Top 100 (LogFC) downregulated transcripts in resistant red maple (*Acer rubrum*) genotypes compared to water controls.**

| **Rank** | **Transcript ID** | **Plants (RPKM)** | | | | | | **LogFC** | **Adj. P.Value** | **Description** |
| --- | --- | --- | --- | --- | --- | --- | --- | --- | --- | --- |
|  |  | **Res. 1** | **Res. 2** | **Res. 3** | **H_2_O 1** | **H_2_O 2** | **H_2_O 3** |  |  |  |
| 1 | TRINITY_DN438303_c0_g1 | 0.00 | 0.00 | 0.00 | 32.37 | 51.81 | 54.69 | -10.70 | 0.00208 | Purple acid phosphatase |
| 2 | TRINITY_DN429524_c0_g2 | 0.00 | 0.00 | 0.00 | 13.80 | 29.16 | 111.83 | -9.17 | 0.00666 | Putative protein LURP-one-related 10-like |
| 3 | TRINITY_DN439956_c0_g1 | 0.00 | 0.00 | 0.00 | 123.28 | 50.92 | 115.24 | -8.93 | 0.00208 | ACD1-like |
| 4 | TRINITY_DN437970_c0_g1 | 0.00 | 0.00 | 0.58 | 58.15 | 27.49 | 41.18 | -8.89 | 0.00649 | Putative cyclin B1 |
| 5 | TRINITY_DN442867_c0_g1 | 0.00 | 0.00 | 0.00 | 50.14 | 32.79 | 8.70 | -8.82 | 0.00388 | Patatin |
| 6 | TRINITY_DN425288_c0_g1 | 0.00 | 0.00 | 0.00 | 167.32 | 20.03 | 213.29 | -8.77 | 0.01273 | Putative organ-specific protein S2-like |
| 7 | TRINITY_DN422431_c1_g1 | 0.00 | 0.00 | 0.00 | 97.09 | 7.83 | 180.75 | -8.74 | 0.02982 | Putative organ-specific protein P4-like isoform X2 |
| 8 | TRINITY_DN431193_c1_g1 | 0.00 | 0.00 | 0.00 | 166.63 | 45.63 | 41.28 | -8.73 | 0.00273 | Putative 14 kDa proline-rich protein DC2.15 |
| 9 | TRINITY_DN433979_c0_g1 | 0.00 | 0.00 | 0.00 | 9.98 | 25.74 | 16.79 | -8.62 | 0.00208 | AT3g20370/MQC12_13 |
| 10 | TRINITY_DN428010_c1_g1 | 0.00 | 0.00 | 0.00 | 40.35 | 6.11 | 8.93 | -8.61 | 0.00523 | Lipoxygenase |
| 11 | TRINITY_DN429852_c0_g1 | 0.00 | 0.24 | 0.66 | 34.20 | 75.64 | 84.42 | -8.43 | 0.02260 | Sulfotransferase |
| 12 | TRINITY_DN435667_c3_g1 | 0.11 | 3.94 | 3.92 | 591.62 | 391.57 | 544.19 | -8.41 | 0.01602 | Histone H2A |
| 13 | TRINITY_DN438161_c2_g3 | 0.00 | 0.00 | 0.08 | 19.43 | 18.62 | 15.06 | -8.40 | 0.00208 | Putative sphingolipid delta 4 desaturase/C-4 hydroxylase protein des2 |
| 14 | TRINITY_DN438742_c0_g1 | 0.00 | 0.00 | 0.00 | 10.33 | 10.13 | 14.72 | -8.39 | 0.00208 | PHD finger family protein |
| 15 | TRINITY_DN433832_c1_g1 | 0.00 | 0.00 | 0.00 | 136.44 | 20.03 | 144.35 | -8.33 | 0.00208 | Predicted protein |
| 16 | TRINITY_DN430711_c1_g1 | 0.00 | 0.00 | 0.00 | 24.35 | 34.47 | 25.46 | -8.30 | 0.00208 | Pectinesterase |
| 17 | TRINITY_DN434996_c3_g3 | 0.00 | 0.07 | 0.00 | 14.26 | 8.59 | 16.56 | -8.27 | 0.00212 | Coffea canephora DH200=94 genomic scaffold |
| 18 | TRINITY_DN441538_c0_g2 | 0.00 | 0.00 | 0.00 | 237.51 | 326.15 | 163.70 | -8.22 | 0.00208 | Putative flavonol synthase/flavanone 3-hydroxylase |
| 19 | TRINITY_DN440736_c1_g2 | 0.13 | 0.00 | 0.00 | 34.77 | 9.74 | 36.29 | -8.21 | 0.00307 | Expansin B3, BETA 1.6 isoform 1 |
| 20 | TRINITY_DN442303_c1_g3 | 0.00 | 0.00 | 0.00 | 18.16 | 5.98 | 30.21 | -8.20 | 0.00209 | Cyclin family protein |
| 21 | TRINITY_DN441216_c1_g2 | 0.00 | 1.07 | 1.65 | 106.58 | 76.52 | 112.14 | -8.19 | 0.00683 | Leucoanthocyanidin reductase |
| 22 | TRINITY_DN441930_c4_g8 | 0.00 | 0.00 | 0.00 | 26.10 | 14.91 | 13.68 | -8.17 | 0.00208 | PREDICTED: epidermis-specific secreted glycoprotein EP1-like |
| 23 | TRINITY_DN435341_c0_g1 | 0.00 | 0.00 | 2.08 | 84.71 | 21.56 | 42.05 | -8.15 | 0.04806 | Peroxidase |
| 24 | TRINITY_DN432222_c0_g2 | 0.00 | 0.48 | 10.67 | 160.25 | 160.56 | 137.79 | -8.15 | 0.00807 | 4-coumarate:CoA ligase 3 |
| 25 | TRINITY_DN441888_c1_g2 | 0.00 | 0.00 | 0.75 | 31.98 | 13.24 | 21.72 | -8.06 | 0.00733 | Putative alpha-l-fucosidase |
| 26 | TRINITY_DN439841_c1_g1 | 0.00 | 0.00 | 0.00 | 20.83 | 7.51 | 7.38 | -7.98 | 0.00210 | Putative carboxyesterase 20 |
| 27 | TRINITY_DN443309_c1_g1 | 0.00 | 0.00 | 0.00 | 27.91 | 13.24 | 35.86 | -7.95 | 0.00208 | Coffea canephora DH200=94 genomic scaffold |
| 28 | TRINITY_DN446489_c0_g1 | 0.00 | 0.00 | 0.00 | 21.43 | 12.72 | 6.95 | -7.95 | 0.00208 | Putative CRINKLY4 related 3 |
| 29 | TRINITY_DN451023_c1_g5 | 0.00 | 0.00 | 0.00 | 127.59 | 89.40 | 95.45 | -7.94 | 0.00208 | Putative glucan endo-1,3-beta-glucosidase 12 |
| 30 | TRINITY_DN446485_c1_g1 | 0.00 | 0.00 | 0.64 | 31.33 | 45.83 | 45.39 | -7.93 | 0.00276 | RNA polymerase II elongation factor ELL3 isoform 1 |
| 31 | TRINITY_DN446531_c1_g1 | 0.00 | 6.00 | 0.00 | 55.56 | 14.89 | 28.46 | -7.90 | 0.02332 | Lipoxygenase |
| 32 | TRINITY_DN437761_c0_g1 | 0.00 | 0.00 | 0.00 | 10.44 | 6.87 | 8.08 | -7.87 | 0.00208 | Pectinesterase |
| 33 | TRINITY_DN433596_c0_g1 | 0.00 | 0.00 | 0.00 | 14.62 | 6.26 | 3.84 | -7.86 | 0.00230 | Xyloglucan endotransglucosylase/hydrolase |
| 34 | TRINITY_DN428390_c0_g1 | 0.00 | 11.78 | 2.83 | 265.87 | 398.14 | 364.21 | -7.84 | 0.01040 | Xyloglucan endotransglucosylase/hydrolase |
| 35 | TRINITY_DN429559_c0_g2 | 0.00 | 0.00 | 0.00 | 11.45 | 22.37 | 23.11 | -7.84 | 0.00208 | Putative RNA-binding protein 27 |
| 36 | TRINITY_DN438813_c1_g2 | 2.81 | 6.68 | 74.36 | 1991.69 | 2509.13 | 2868.16 | -7.84 | 0.00630 | Putative organ-specific protein S2-like |
| 37 | TRINITY_DN429522_c0_g1 | 0.00 | 0.00 | 0.00 | 35.97 | 23.61 | 10.57 | -7.83 | 0.00208 | Putative Annexin 8 |
| 38 | TRINITY_DN432177_c1_g1 | 1.00 | 0.47 | 0.00 | 111.89 | 59.13 | 75.99 | -7.82 | 0.01474 | Actin-97 |
| 39 | TRINITY_DN429585_c3_g1 | 0.00 | 0.00 | 0.74 | 19.31 | 33.99 | 22.16 | -7.82 | 0.00446 | Putative protein PPLZ02 |
| 40 | TRINITY_DN432192_c0_g1 | 0.00 | 0.00 | 0.00 | 60.40 | 10.88 | 37.88 | -7.79 | 0.00336 | Putative TRNA Delta(2)-isopentenylpyrophosphate transferase |
| 41 | TRINITY_DN436295_c2_g6 | 0.00 | 0.00 | 0.00 | 42.90 | 23.40 | 12.78 | -7.77 | 0.00223 | Putative wall-associated receptor kinase 2-like |
| 42 | TRINITY_DN450830_c0_g1 | 0.00 | 0.00 | 0.00 | 29.50 | 3.39 | 5.45 | -7.75 | 0.00662 | Allergen Act d 3 |
| 43 | TRINITY_DN427242_c0_g1 | 0.17 | 5.08 | 20.64 | 509.71 | 1023.68 | 481.96 | -7.71 | 0.02903 | Putative cytochrome P450 71D10 |
| 44 | TRINITY_DN439656_c0_g3 | 0.00 | 0.16 | 0.00 | 21.44 | 33.05 | 10.48 | -7.68 | 0.00318 | Pectin lyase-like superfamily protein isoform 1 |
| 45 | TRINITY_DN441310_c0_g1 | 0.00 | 1.40 | 1.78 | 94.64 | 44.74 | 103.94 | -7.67 | 0.04085 | Syntaxin of plants 111 |
| 46 | TRINITY_DN439413_c0_g1 | 0.00 | 0.00 | 0.00 | 18.85 | 16.18 | 3.92 | -7.65 | 0.00302 | Putative steroid dehydrogenase |
| 47 | TRINITY_DN415915_c0_g2 | 0.00 | 0.00 | 0.00 | 24.99 | 15.01 | 11.65 | -7.63 | 0.00208 | NA |
| 48 | TRINITY_DN437153_c1_g1 | 0.05 | 2.77 | 0.00 | 13.30 | 29.25 | 20.20 | -7.61 | 0.00390 | DNA primase |
| 49 | TRINITY_DN440088_c0_g4 | 0.00 | 0.00 | 4.31 | 233.80 | 102.87 | 113.58 | -7.61 | 0.00871 | Putative peamaclein-like |
| 50 | TRINITY_DN445462_c0_g1 | 0.00 | 0.00 | 0.69 | 18.94 | 9.06 | 13.69 | -7.60 | 0.00756 | Putative axoneme-associated protein mst101(2)-like |
| 51 | TRINITY_DN433129_c0_g3 | 0.00 | 0.00 | 1.07 | 19.65 | 51.10 | 9.66 | -7.58 | 0.01317 | DUF594 family protein |
| 52 | TRINITY_DN402874_c0_g1 | 0.00 | 0.00 | 0.00 | 9.65 | 4.45 | 8.18 | -7.56 | 0.00208 | Putative probable rhamnose biosynthetic enzyme 1 |
| 53 | TRINITY_DN441567_c4_g1 | 0.00 | 3.93 | 22.74 | 584.57 | 349.23 | 903.02 | -7.54 | 0.04073 | Histone H3.2 n |
| 54 | TRINITY_DN440303_c0_g12 | 0.00 | 0.00 | 0.00 | 28.65 | 17.17 | 5.94 | -7.53 | 0.00210 | Putative wall-associated receptor kinase 2-like |
| 55 | TRINITY_DN426113_c0_g1 | 0.00 | 0.00 | 0.00 | 27.97 | 11.77 | 20.07 | -7.48 | 0.00208 | Coffea canephora DH200=94 genomic scaffold |
| 56 | TRINITY_DN436501_c1_g1 | 0.00 | 0.00 | 0.00 | 33.70 | 46.97 | 65.28 | -7.46 | 0.00208 | Putative cell wall integrity and stress response component 2 |
| 57 | TRINITY_DN429116_c2_g2 | 0.21 | 0.26 | 0.00 | 63.98 | 26.10 | 55.57 | -7.45 | 0.00433 | Ankyrin repeat family protein |
| 58 | TRINITY_DN435058_c3_g1 | 0.00 | 0.00 | 0.78 | 28.98 | 50.96 | 16.49 | -7.44 | 0.00739 | Putative protein trichome birefringence-like 41 |
| 59 | TRINITY_DN433502_c0_g1 | 0.00 | 0.56 | 29.32 | 273.04 | 201.52 | 154.76 | -7.44 | 0.00913 | Laccase |
| 60 | TRINITY_DN434426_c0_g1 | 0.24 | 0.00 | 0.00 | 24.88 | 6.37 | 3.08 | -7.43 | 0.03569 | Cuperdoxin domain |
| 61 | TRINITY_DN443119_c3_g1 | 0.00 | 1.17 | 0.00 | 38.12 | 13.88 | 18.24 | -7.43 | 0.02081 | Cytochrome P450 |
| 62 | TRINITY_DN445364_c1_g3 | 0.00 | 0.00 | 1.37 | 117.95 | 54.98 | 61.49 | -7.41 | 0.00417 | Pectate lyase |
| 63 | TRINITY_DN427192_c0_g1 | 0.00 | 0.00 | 0.00 | 25.04 | 13.77 | 7.51 | -7.38 | 0.00220 | Transcription factor bHLH61-like protein |
| 64 | TRINITY_DN442004_c0_g1 | 0.00 | 1.45 | 0.00 | 34.52 | 27.61 | 16.09 | -7.36 | 0.01136 | Putative shikimate dehydrogenase |
| 65 | TRINITY_DN432103_c1_g1 | 0.00 | 0.00 | 0.00 | 3.35 | 4.50 | 2.64 | -7.35 | 0.00208 | Putative inactive leucine-rich repeat receptor-like protein kinase At3g03770 isoform X1 |
| 66 | TRINITY_DN439652_c1_g1 | 0.00 | 0.00 | 0.07 | 61.34 | 4.38 | 28.61 | -7.33 | 0.00350 | Lysophosphatidyl acyltransferase 5 |
| 67 | TRINITY_DN434313_c0_g3 | 0.00 | 0.40 | 0.09 | 27.96 | 14.49 | 26.52 | -7.33 | 0.00742 | Putative TPX2 protein family |
| 68 | TRINITY_DN438667_c0_g1 | 0.00 | 0.00 | 0.00 | 6.17 | 8.17 | 4.87 | -7.32 | 0.00208 | DNA double-strand break repair rad50 ATPase, putative isoform 1 |
| 69 | TRINITY_DN440836_c0_g2 | 2.10 | 38.23 | 71.81 | 2786.95 | 2555.61 | 2294.85 | -7.32 | 0.00433 | Putative cytochrome B5 isoform D-like |
| 70 | TRINITY_DN425318_c1_g1 | 6.34 | 0.00 | 0.00 | 198.57 | 54.09 | 101.06 | -7.31 | 0.02828 | Actin-7 |
| 71 | TRINITY_DN436787_c4_g2 | 0.00 | 0.45 | 0.56 | 27.74 | 22.67 | 34.88 | -7.31 | 0.00825 | Fatty acid/sphingolipid desaturase |
| 72 | TRINITY_DN440779_c1_g2 | 0.00 | 4.88 | 0.00 | 303.98 | 857.13 | 599.77 | -7.29 | 0.00439 | Anthocyanidin reductase |
| 73 | TRINITY_DN443005_c2_g1 | 0.09 | 0.00 | 2.09 | 44.77 | 22.43 | 55.71 | -7.27 | 0.01505 | Peroxidase |
| 74 | TRINITY_DN442153_c0_g3 | 0.00 | 5.93 | 2.02 | 255.20 | 129.51 | 271.16 | -7.26 | 0.02253 | Predicted high mobility group B protein 7 n |
| 75 | TRINITY_DN424299_c0_g2 | 0.00 | 0.30 | 0.00 | 7.10 | 26.20 | 37.80 | -7.26 | 0.01174 | Predicted metalloendoproteinase 1-like |
| 76 | TRINITY_DN435384_c0_g1 | 0.00 | 0.09 | 0.42 | 31.58 | 9.32 | 23.30 | -7.25 | 0.02101 | Cyclin family protein |
| 77 | TRINITY_DN442864_c3_g2 | 1.30 | 6.60 | 23.72 | 771.96 | 1047.12 | 681.58 | -7.25 | 0.00208 | Chalcone synthase |
| 78 | TRINITY_DN432607_c1_g1 | 0.00 | 0.00 | 4.17 | 57.60 | 42.73 | 37.96 | -7.25 | 0.00871 | Hydroxyproline-rich glycoprotein family protein |
| 79 | TRINITY_DN431553_c0_g2 | 0.00 | 0.00 | 0.00 | 59.35 | 21.91 | 111.03 | -7.25 | 0.00328 | Putative calcium-binding EF-hand family protein |
| 80 | TRINITY_DN435667_c2_g2 | 0.00 | 0.00 | 0.00 | 11.80 | 2.83 | 13.53 | -7.24 | 0.00208 | Pectinesterase |
| 81 | TRINITY_DN436184_c3_g2 | 0.00 | 0.00 | 1.04 | 26.56 | 12.92 | 8.69 | -7.22 | 0.01897 | Cytochrome P450 |
| 82 | TRINITY_DN447448_c2_g1 | 2.14 | 0.00 | 0.06 | 19.47 | 19.59 | 32.64 | -7.21 | 0.01255 | Putative boron transporter |
| 83 | TRINITY_DN437779_c3_g1 | 0.00 | 0.17 | 0.85 | 50.83 | 18.88 | 30.37 | -7.19 | 0.02374 | Beta glucosidase 46 isoform 1 |
| 84 | TRINITY_DN446037_c2_g2 | 0.00 | 0.00 | 4.81 | 164.64 | 33.30 | 88.45 | -7.19 | 0.03930 | Caffeic acid 3-O-methyltransferase |
| 85 | TRINITY_DN444245_c2_g3 | 0.00 | 1.17 | 1.66 | 55.33 | 49.80 | 65.32 | -7.19 | 0.01103 | Reticulon-like protein |
| 86 | TRINITY_DN441415_c1_g1 | 0.00 | 13.91 | 0.00 | 719.09 | 691.91 | 1100.77 | -7.19 | 0.00578 | Amino acid permease 2 isoform 1 |
| 87 | TRINITY_DN436802_c2_g5 | 0.00 | 0.00 | 0.00 | 21.61 | 4.54 | 5.54 | -7.19 | 0.00301 | Acyl-CoA dehydrogenase |
| 88 | TRINITY_DN431702_c1_g1 | 0.00 | 0.00 | 0.07 | 11.30 | 7.37 | 9.56 | -7.17 | 0.00208 | Putative cytochrome P450 71A9-like |
| 89 | TRINITY_DN428549_c0_g1 | 0.00 | 4.49 | 12.21 | 218.88 | 162.64 | 227.08 | -7.17 | 0.01619 | Histone H2A |
| 90 | TRINITY_DN435214_c0_g1 | 0.00 | 0.00 | 0.00 | 8.70 | 5.22 | 3.86 | -7.13 | 0.00208 | Histidine--tRNA ligase |
| 91 | TRINITY_DN440654_c0_g2 | 0.29 | 0.00 | 0.00 | 12.57 | 6.16 | 11.27 | -7.13 | 0.00621 | Portal 56 |
| 92 | TRINITY_DN447490_c0_g3 | 0.00 | 0.00 | 1.20 | 20.16 | 9.42 | 11.77 | -7.12 | 0.01746 | Kinesin-like protein |
| 93 | TRINITY_DN447210_c0_g5 | 0.00 | 0.00 | 0.17 | 5.75 | 6.19 | 14.08 | -7.11 | 0.00328 | Leucine-rich repeat protein kinase family protein isoform 1 |
| 94 | TRINITY_DN438063_c0_g1 | 0.00 | 0.00 | 0.00 | 4.36 | 17.42 | 7.07 | -7.11 | 0.00230 | Xyloglucan endotransglucosylase/hydrolase |
| 95 | TRINITY_DN439326_c0_g9 | 0.00 | 0.51 | 0.00 | 48.48 | 64.94 | 35.60 | -7.10 | 0.00232 | NA |
| 96 | TRINITY_DN442736_c0_g1 | 0.00 | 0.00 | 0.00 | 15.15 | 10.54 | 8.02 | -7.09 | 0.00208 | Predicted protein |
| 97 | TRINITY_DN430929_c2_g1 | 0.00 | 0.00 | 0.00 | 12.19 | 4.68 | 12.71 | -7.09 | 0.00230 | Putative S-adenosyl-L-methionine-dependent methyltransferases superfamily protein |
| 98 | TRINITY_DN443184_c2_g1 | 0.00 | 0.51 | 0.00 | 43.49 | 29.81 | 58.57 | -7.09 | 0.00980 | Putative carboxy-lyase |
| 99 | TRINITY_DN434885_c0_g1 | 0.00 | 0.00 | 0.18 | 9.48 | 2.41 | 11.92 | -7.08 | 0.00722 | Threonine dehydratase |
| 100 | TRINITY_DN442279_c1_g1 | 0.00 | 0.06 | 1.72 | 25.49 | 13.97 | 14.95 | -7.08 | 0.01878 | Scarecrow-like protein 28 |

**Table 3S. Top 100 (LogFC) upregulated transcripts in susceptible red maple (*Acer rubrum*) genotypes compared to water controls.**

| **Rank** | **Transcript ID** | **Plants (RPKM)** | | | | | **LogFC** | | **Adj. P.Value** | | **Description** | |  |
| --- | --- | --- | --- | --- | --- | --- | --- | --- | --- | --- | --- | --- | --- |
|  |  | **Sus. 1** | **Sus. 2** | **H_2_O 1** | **H_2_O 2** | **H_2_O 3** | |  | |  | |  | |
| 1 | TRINITY_DN426651_c2_g1 | 22.77 | 344.72 | 0.00 | 0.00 | 0.00 | | 10.43 | | 0.04809 | | Alkaline proteinase | |
| 2 | TRINITY_DN432097_c0_g1 | 33.34 | 25.00 | 0.00 | 0.00 | 0.00 | | 9.98 | | 0.00831 | | Chloride channel protein | |
| 3 | TRINITY_DN435926_c1_g6 | 540.37 | 510.08 | 0.00 | 0.00 | 0.00 | | 9.96 | | 0.00831 | | DCD domain protein isoform 1 | |
| 4 | TRINITY_DN449241_c4_g3 | 439.64 | 460.82 | 0.00 | 0.00 | 11.67 | | 9.90 | | 0.02447 | | Putative non-symbiotic hemoglobin | |
| 5 | TRINITY_DN446102_c0_g1 | 376.84 | 172.26 | 0.00 | 0.00 | 0.00 | | 9.80 | | 0.00831 | | Putative glycine-rich protein DC7.1 | |
| 6 | TRINITY_DN375151_c0_g1 | 73.50 | 25.73 | 0.00 | 0.00 | 0.00 | | 9.79 | | 0.00859 | | 60S ribosomal protein L6 | |
| 7 | TRINITY_DN426807_c0_g1 | 29.20 | 21.24 | 0.00 | 0.00 | 0.00 | | 9.70 | | 0.00831 | | Albugo candida WGS project CAIX00000000 data | |
| 8 | TRINITY_DN428352_c1_g1 | 30.61 | 31.82 | 0.00 | 0.00 | 1.13 | | 9.70 | | 0.02995 | | Hypothetical protein | |
| 9 | TRINITY_DN436977_c0_g1 | 65.96 | 21.78 | 0.00 | 0.00 | 0.00 | | 9.68 | | 0.00881 | | Cyclin p4 | |
| 10 | TRINITY_DN439029_c0_g7 | 318.85 | 353.07 | 0.00 | 0.00 | 0.00 | | 9.67 | | 0.00831 | | NA | |
| 11 | TRINITY_DN446442_c1_g1 | 28.25 | 113.24 | 0.00 | 0.00 | 0.00 | | 9.67 | | 0.01058 | | Non-symbiotic hemoglobin | |
| 12 | TRINITY_DN451117_c1_g2 | 49.99 | 87.39 | 0.00 | 0.00 | 0.00 | | 9.65 | | 0.00831 | | Putative kinase | |
| 13 | TRINITY_DN431010_c0_g1 | 7.49 | 35.22 | 0.00 | 0.00 | 0.00 | | 9.56 | | 0.01218 | | T1.1 protein | |
| 14 | TRINITY_DN445195_c1_g1 | 9.24 | 9.99 | 0.00 | 0.00 | 0.00 | | 9.50 | | 0.00831 | | Putative cucumisin-like | |
| 15 | TRINITY_DN451117_c0_g4 | 106.48 | 178.60 | 0.00 | 0.00 | 0.00 | | 9.31 | | 0.00831 | | Kinase-like protein (Fragment) | |
| 16 | TRINITY_DN329160_c0_g1 | 11.83 | 94.08 | 0.00 | 0.00 | 0.00 | | 9.30 | | 0.01858 | | Putative vegetative cell wall protein gp1-like | |
| 17 | TRINITY_DN428750_c1_g1 | 14.62 | 65.21 | 0.00 | 0.00 | 0.00 | | 9.24 | | 0.00873 | | Putative tonoplast intrinsic protein | |
| 18 | TRINITY_DN459004_c0_g1 | 58.34 | 36.64 | 0.00 | 0.00 | 0.00 | | 9.18 | | 0.00831 | | RpL31_0 protein (Fragment) | |
| 19 | TRINITY_DN443554_c2_g4 | 96.81 | 1735.86 | 0.00 | 0.00 | 0.00 | | 9.16 | | 0.03746 | | Coffea canephora DH200=94 genomic scaffold | |
| 20 | TRINITY_DN448079_c3_g3 | 1478.36 | 695.12 | 0.00 | 0.00 | 0.00 | | 9.15 | | 0.00831 | | Putative TMV resistance protein N-like | |
| 21 | TRINITY_DN464912_c0_g1 | 48.70 | 26.10 | 0.00 | 0.00 | 0.00 | | 9.15 | | 0.00831 | | TSA: Wollemia nobilis Ref_Wollemi_Transcript_25331_947 transcribed RNA sequence | |
| 22 | TRINITY_DN427874_c0_g1 | 221.22 | 86.41 | 0.00 | 2.47 | 0.00 | | 9.13 | | 0.04765 | | Actophorin | |
| 23 | TRINITY_DN472589_c0_g1 | 204.76 | 59.10 | 0.95 | 0.00 | 0.00 | | 9.13 | | 0.04031 | | C12D12.1 isoform c | |
| 24 | TRINITY_DN459296_c0_g1 | 69.67 | 15.96 | 0.00 | 0.00 | 0.00 | | 9.13 | | 0.01085 | | S9 n | |
| 25 | TRINITY_DN470902_c0_g1 | 310.61 | 68.25 | 0.00 | 0.00 | 0.00 | | 9.11 | | 0.01117 | | Putative 40S ribosomal protein S29 | |
| 26 | TRINITY_DN395243_c0_g2 | 6.06 | 54.06 | 0.00 | 0.00 | 0.00 | | 9.11 | | 0.01978 | | Putative basic proline-rich protein-like | |
| 27 | TRINITY_DN467430_c0_g1 | 40.08 | 24.38 | 0.00 | 0.00 | 0.00 | | 9.10 | | 0.00831 | | Golgin subfamily A member 6-like protein 2 | |
| 28 | TRINITY_DN430597_c1_g4 | 47.73 | 85.29 | 0.00 | 0.00 | 0.00 | | 9.09 | | 0.00831 | | Putative polyprotein of LTR transposon | |
| 29 | TRINITY_DN433807_c0_g4 | 22.39 | 17.17 | 0.00 | 0.00 | 0.00 | | 9.05 | | 0.00831 | | 40S ribosomal protein S6 | |
| 30 | TRINITY_DN443516_c1_g1 | 4.36 | 75.06 | 0.00 | 0.00 | 0.00 | | 9.04 | | 0.03226 | | Putative ACT domain-containing protein ACR2 | |
| 31 | TRINITY_DN439596_c1_g1 | 23.31 | 31.83 | 0.00 | 0.00 | 0.00 | | 9.00 | | 0.00831 | | Putative growth factor independence | |
| 32 | TRINITY_DN441088_c1_g2 | 4.95 | 69.98 | 0.00 | 0.00 | 0.00 | | 8.99 | | 0.00888 | | Ankyrin repeat family protein, putative isoform 2 | |
| 33 | TRINITY_DN445930_c2_g1 | 99.66 | 197.42 | 0.00 | 0.00 | 0.72 | | 8.95 | | 0.01418 | | Putative galactinol synthase 2-like | |
| 34 | TRINITY_DN301725_c0_g1 | 60.62 | 23.02 | 0.10 | 0.00 | 0.00 | | 8.93 | | 0.01085 | | 60S ribosomal protein L13a | |
| 35 | TRINITY_DN448801_c2_g1 | 362.30 | 180.71 | 0.00 | 1.11 | 0.73 | | 8.92 | | 0.02510 | | Dehydrin | |
| 36 | TRINITY_DN432002_c5_g5 | 2.65 | 22.92 | 0.00 | 0.00 | 0.00 | | 8.91 | | 0.01887 | | Glucanase | |
| 37 | TRINITY_DN439142_c1_g4 | 198.50 | 1296.66 | 0.00 | 0.00 | 0.00 | | 8.91 | | 0.01529 | | Putative glutathione S-transferase | |
| 38 | TRINITY_DN435281_c0_g1 | 11.05 | 91.08 | 0.00 | 0.00 | 0.00 | | 8.91 | | 0.01816 | | Cellulose synthase | |
| 39 | TRINITY_DN241464_c0_g1 | 8.84 | 29.36 | 0.00 | 0.00 | 0.00 | | 8.91 | | 0.00888 | | Similar to Pc16g11320 [Penicillium chrysogenum Wisconsin 54-1255] acc. no. XP_002561436 | |
| 40 | TRINITY_DN433234_c2_g1 | 9.59 | 3.15 | 0.00 | 0.00 | 0.00 | | 8.91 | | 0.00873 | | Hypothetical protein SAMD00019534_057880 | |
| 41 | TRINITY_DN438880_c2_g2 | 33.56 | 21.91 | 0.08 | 0.00 | 0.07 | | 8.90 | | 0.01136 | | Endoglucanase | |
| 42 | TRINITY_DN437658_c0_g1 | 16.86 | 14.95 | 0.00 | 0.00 | 0.00 | | 8.87 | | 0.00831 | | EF hand domain protein | |
| 43 | TRINITY_DN464773_c0_g1 | 42.10 | 23.74 | 0.36 | 0.00 | 0.00 | | 8.84 | | 0.01715 | | NADH-ubiquinone oxidoreductase chain 4 (Fragment) | |
| 44 | TRINITY_DN437425_c6_g3 | 25.35 | 59.52 | 0.12 | 0.00 | 0.00 | | 8.84 | | 0.01250 | | Peptidyl-prolyl cis-trans isomerase | |
| 45 | TRINITY_DN387408_c0_g1 | 40.36 | 22.70 | 0.00 | 0.00 | 0.00 | | 8.81 | | 0.00831 | | 40S ribosomal protein S18 | |
| 46 | TRINITY_DN89056_c0_g2 | 93.96 | 26.10 | 0.00 | 0.00 | 0.44 | | 8.80 | | 0.04177 | | NA | |
| 47 | TRINITY_DN439958_c7_g5 | 27.04 | 14.49 | 0.00 | 0.06 | 0.00 | | 8.80 | | 0.00881 | | Receptor of activated protein kinase C 1A, component of 40S small ribosomal subunit | |
| 48 | TRINITY_DN431524_c1_g1 | 259.73 | 106.58 | 0.00 | 0.00 | 0.00 | | 8.78 | | 0.00831 | | Major actin | |
| 49 | TRINITY_DN19823_c0_g1 | 36.13 | 36.02 | 0.00 | 0.00 | 0.00 | | 8.76 | | 0.00831 | | Predicted protein | |
| 50 | TRINITY_DN444759_c1_g3 | 1534.75 | 1055.02 | 0.00 | 0.00 | 36.13 | | 8.73 | | 0.01621 | | DUF3774 domain protein | |
| 51 | TRINITY_DN394927_c0_g2 | 49.57 | 23.98 | 0.00 | 0.00 | 0.00 | | 8.72 | | 0.00831 | | Ribosomal protein S20, component of cytosolic 80S ribosome and 40S small subunit | |
| 52 | TRINITY_DN405517_c0_g2 | 41.47 | 23.65 | 0.00 | 0.00 | 0.00 | | 8.69 | | 0.00831 | | Putative 40S ribosomal protein S15 isoform 1 | |
| 53 | TRINITY_DN467540_c0_g1 | 38.04 | 19.43 | 0.00 | 0.00 | 0.00 | | 8.68 | | 0.00831 | | Putative basic proline-rich protein-like | |
| 54 | TRINITY_DN448785_c3_g12 | 265.87 | 1468.16 | 0.00 | 0.00 | 0.00 | | 8.68 | | 0.01314 | | Monodehydroascorbate reductase family protein | |
| 55 | TRINITY_DN142237_c0_g1 | 36.14 | 22.02 | 0.15 | 0.00 | 0.00 | | 8.67 | | 0.00969 | | Ribosomal protein | |
| 56 | TRINITY_DN434811_c6_g1 | 4.63 | 54.50 | 0.00 | 0.00 | 0.00 | | 8.66 | | 0.02337 | | Epsilon frustilin | |
| 57 | TRINITY_DN442555_c0_g1 | 9.36 | 40.60 | 0.00 | 0.08 | 0.00 | | 8.65 | | 0.02511 | | Putative inactive poly [ADP-ribose] polymerase SRO2 | |
| 58 | TRINITY_DN470663_c0_g1 | 40.62 | 19.13 | 0.00 | 0.00 | 0.00 | | 8.64 | | 0.00831 | | 50S ribosomal protein L34e | |
| 59 | TRINITY_DN429635_c5_g1 | 13.32 | 8.72 | 0.00 | 0.00 | 0.00 | | 8.63 | | 0.00831 | | Peroxisomal ascorbate peroxidase | |
| 60 | TRINITY_DN453501_c0_g1 | 9.17 | 5.31 | 0.00 | 0.00 | 0.00 | | 8.62 | | 0.00831 | | Putative tripeptidyl-peptidase 1 | |
| 61 | TRINITY_DN426185_c1_g2 | 1.71 | 47.05 | 0.00 | 0.00 | 0.00 | | 8.62 | | 0.04849 | | GPI anchored serine-rich protein | |
| 62 | TRINITY_DN439256_c5_g5 | 141.24 | 198.81 | 0.00 | 0.00 | 1.46 | | 8.61 | | 0.01234 | | NA | |
| 63 | TRINITY_DN320648_c0_g1 | 43.11 | 13.35 | 0.00 | 0.00 | 0.00 | | 8.61 | | 0.00881 | | 40S ribosomal protein S14 (Fragment) | |
| 64 | TRINITY_DN425980_c1_g2 | 1.19 | 30.06 | 0.00 | 0.00 | 0.00 | | 8.59 | | 0.04257 | | Cell wall protein | |
| 65 | TRINITY_DN450014_c0_g2 | 2.83 | 28.74 | 0.00 | 0.00 | 0.00 | | 8.58 | | 0.02066 | | Extracellular serine-threonine rich protein | |
| 66 | TRINITY_DN15284_c0_g2 | 18.26 | 8.49 | 0.00 | 0.00 | 0.00 | | 8.57 | | 0.00831 | | FAM49 family protein | |
| 67 | TRINITY_DN427672_c0_g1 | 18.13 | 8.19 | 0.00 | 0.00 | 0.00 | | 8.56 | | 0.00831 | | Chloride channel protein | |
| 68 | TRINITY_DN373295_c0_g2 | 14.59 | 89.46 | 0.00 | 0.00 | 0.00 | | 8.56 | | 0.01405 | | Predicted protein | |
| 69 | TRINITY_DN436844_c0_g1 | 2.27 | 74.65 | 0.00 | 0.00 | 0.00 | | 8.56 | | 0.04989 | | Predicted protein | |
| 70 | TRINITY_DN427425_c8_g1 | 2.73 | 21.53 | 0.00 | 0.00 | 0.00 | | 8.53 | | 0.01716 | | Endoglucanase | |
| 71 | TRINITY_DN470049_c0_g1 | 20.51 | 17.60 | 0.00 | 0.00 | 0.00 | | 8.52 | | 0.00831 | | Rab GTPase | |
| 72 | TRINITY_DN467452_c0_g1 | 21.03 | 9.52 | 0.00 | 0.00 | 0.00 | | 8.51 | | 0.00831 | | PREDICTED: collagen alpha-1(XII) chain-like | |
| 73 | TRINITY_DN445790_c0_g3 | 18.45 | 49.46 | 0.00 | 0.00 | 0.00 | | 8.50 | | 0.00852 | | Chloroplast RNA-binding protein 33 | |
| 74 | TRINITY_DN446519_c2_g3 | 22.35 | 59.80 | 0.13 | 0.00 | 0.00 | | 8.49 | | 0.01141 | | Putative olfactory receptor 7A2 | |
| 75 | TRINITY_DN122733_c0_g1 | 73.45 | 45.94 | 0.00 | 0.00 | 0.24 | | 8.49 | | 0.00888 | | Putative ribosomal protein L35 | |
| 76 | TRINITY_DN463178_c0_g1 | 3.06 | 40.50 | 0.00 | 0.00 | 0.00 | | 8.47 | | 0.02524 | | Cutinase | |
| 77 | TRINITY_DN380536_c1_g3 | 2.98 | 39.81 | 0.00 | 0.00 | 0.00 | | 8.47 | | 0.02551 | | Putative lipase esterase family protein | |
| 78 | TRINITY_DN447378_c2_g1 | 8.59 | 11.66 | 0.00 | 0.00 | 0.00 | | 8.46 | | 0.00831 | | Glycosyltransferase | |
| 79 | TRINITY_DN448079_c3_g1 | 123.43 | 229.20 | 1.64 | 0.00 | 0.00 | | 8.46 | | 0.01218 | | Putative TMV resistance protein N-like | |
| 80 | TRINITY_DN445930_c2_g4 | 36.04 | 124.64 | 0.00 | 0.00 | 0.00 | | 8.46 | | 0.00947 | | Putative galactinol synthase 1-like | |
| 81 | TRINITY_DN377099_c0_g1 | 105.24 | 49.96 | 0.28 | 0.00 | 0.00 | | 8.43 | | 0.00941 | | 40S ribosomal protein S27 | |
| 8 | TRINITY_DN438738_c1_g1 | 53.30 | 55.73 | 1.28 | 0.00 | 0.00 | | 8.43 | | 0.01933 | | 60S ribosomal protein L20 | |
| 83 | TRINITY_DN470840_c0_g1 | 21.02 | 10.62 | 0.05 | 0.00 | 0.00 | | 8.40 | | 0.00888 | | Putative cyclic AMP-dependent transcription factor ATF-5 boliviensis boliviensis | |
| 84 | TRINITY_DN40889_c0_g1 | 56.28 | 34.59 | 1.31 | 0.00 | 0.00 | | 8.38 | | 0.02652 | | Predicted protein | |
| 85 | TRINITY_DN446776_c1_g1 | 8.28 | 29.50 | 0.00 | 0.00 | 0.00 | | 8.37 | | 0.00969 | | 4-coumarate:coenzyme a ligase | |
| 86 | TRINITY_DN243883_c0_g3 | 20.38 | 13.74 | 0.00 | 0.00 | 0.00 | | 8.35 | | 0.00831 | | NA | |
| 87 | TRINITY_DN462510_c0_g1 | 93.49 | 59.49 | 0.00 | 0.00 | 0.00 | | 8.33 | | 0.00831 | | P-loop containing nucleoside triphosphate hydrolase protein | |
| 88 | TRINITY_DN387993_c0_g1 | 59.76 | 8.89 | 0.00 | 0.00 | 0.00 | | 8.32 | | 0.01154 | | 60S ribosomal protein L27 | |
| 89 | TRINITY_DN439915_c2_g3 | 3440.07 | 2410.59 | 0.00 | 0.00 | 0.00 | | 8.30 | | 0.00831 | | NA | |
| 90 | TRINITY_DN357949_c0_g1 | 10.91 | 7.23 | 0.00 | 0.00 | 0.00 | | 8.29 | | 0.00831 | | Probable serine/threonine-protein kinase MARK-C | |
| 91 | TRINITY_DN430574_c3_g1 | 3.72 | 30.34 | 0.00 | 0.00 | 0.00 | | 8.28 | | 0.01645 | | Polyprotein | |
| 92 | TRINITY_DN472596_c0_g1 | 43.20 | 27.05 | 0.00 | 0.00 | 0.00 | | 8.24 | | 0.00831 | | Actin-related protein 2/3 complex subunit 5 | |
| 93 | TRINITY_DN442114_c1_g1 | 16.88 | 15.81 | 0.00 | 0.00 | 0.00 | | 8.24 | | 0.00831 | | Ras-related protein Rab-8A | |
| 94 | TRINITY_DN406583_c0_g2 | 18.68 | 6.01 | 0.00 | 0.28 | 0.00 | | 8.24 | | 0.04765 | | High molecular weight heat shock protein | |
| 95 | TRINITY_DN430166_c4_g1 | 1043.06 | 190.90 | 0.00 | 0.00 | 0.00 | | 8.23 | | 0.01253 | | Putative salivary protein (Fragment) | |
| 96 | TRINITY_DN374776_c0_g1 | 3.83 | 22.32 | 0.00 | 0.00 | 0.00 | | 8.23 | | 0.01347 | | Putative extracellular matrix protein | |
| 97 | TRINITY_DN385340_c0_g1 | 1.94 | 15.42 | 0.00 | 0.00 | 0.00 | | 8.23 | | 0.01716 | | Fungal cellulose binding domain-containing protein | |
| 98 | TRINITY_DN469536_c0_g1 | 47.01 | 61.04 | 0.00 | 0.00 | 0.00 | | 8.22 | | 0.00831 | | 60S ribosomal protein L29 | |
| 99 | TRINITY_DN375382_c0_g1 | 2.02 | 20.42 | 0.00 | 0.00 | 0.00 | | 8.22 | | 0.02404 | | Ring finger domain-containing protein | |
| 100 | TRINITY_DN444227_c2_g1 | 21.46 | 4.95 | 0.00 | 0.00 | 0.00 | | 8.20 | | 0.01085 | | 60S acidic ribosomal protein P0 | |

**Table 4S. Top 100 (LogFC) downregulated transcripts in susceptible red maple (*Acer rubrum*) genotypes compared to water controls.**

| **Rank** | **Transcript ID** | **Plants (RPKM)** | | | | | **LogFC** | **Adj. P.Value** | **Description** |
| --- | --- | --- | --- | --- | --- | --- | --- | --- | --- |
|  |  | **Res. 1** | **Res. 2** | **Water 1** | **Water 2** | **Water 3** |  |  |  |
| 1 | TRINITY_DN432222_c0_g2 | 0.00 | 0.00 | 160.25 | 160.56 | 137.79 | -12.39 | 0.00831 | 4-coumarate:CoA ligase 3 |
| 2 | TRINITY_DN440779_c1_g3 | 0.00 | 0.00 | 325.33 | 515.61 | 339.04 | -12.30 | 0.00831 | Anthocyanidin reductase |
| 3 | TRINITY_DN440428_c2_g2 | 0.00 | 0.00 | 87.10 | 73.13 | 56.58 | -11.82 | 0.00831 | Putative AMP dependent CoA ligase |
| 4 | TRINITY_DN441216_c1_g2 | 0.00 | 0.00 | 106.58 | 76.52 | 112.14 | -11.50 | 0.00831 | Leucoanthocyanidin reductase |
| 5 | TRINITY_DN429852_c0_g1 | 0.00 | 0.00 | 34.20 | 75.64 | 84.42 | -11.29 | 0.01314 | Sulfotransferase |
| 6 | TRINITY_DN438303_c0_g1 | 0.00 | 0.00 | 32.37 | 51.81 | 54.69 | -11.22 | 0.00831 | Purple acid phosphatase |
| 7 | TRINITY_DN444245_c2_g3 | 0.00 | 0.00 | 55.33 | 49.80 | 65.32 | -11.18 | 0.00831 | Reticulon-like protein |
| 8 | TRINITY_DN447857_c0_g4 | 0.00 | 0.00 | 36.32 | 10.16 | 19.42 | -10.96 | 0.01573 | Cellulose synthase-like protein D1 |
| 9 | TRINITY_DN443586_c4_g3 | 0.00 | 0.00 | 440.61 | 534.18 | 340.78 | -10.89 | 0.00831 | Copper transporter |
| 10 | TRINITY_DN433607_c0_g1 | 0.15 | 0.00 | 94.48 | 168.31 | 69.59 | -10.86 | 0.04556 | O-methyltransferase |
| 11 | TRINITY_DN430221_c0_g1 | 0.00 | 0.00 | 78.81 | 68.61 | 73.84 | -10.79 | 0.00831 | Limonene synthase |
| 12 | TRINITY_DN434133_c0_g3 | 0.00 | 0.00 | 2759.69 | 7358.98 | 3725.59 | -10.72 | 0.01045 | Coffea canephora DH200=94 genomic scaffold |
| 13 | TRINITY_DN430737_c1_g1 | 0.00 | 0.00 | 49.89 | 49.63 | 56.06 | -10.64 | 0.00831 | Cupredoxin superfamily protein isoform 1 |
| 14 | TRINITY_DN437199_c0_g1 | 0.00 | 0.00 | 34.95 | 12.27 | 23.33 | -10.60 | 0.01425 | Leucine-rich repeat-containing protein 50 |
| 15 | TRINITY_DN437713_c1_g1 | 0.00 | 0.00 | 58.43 | 15.11 | 66.01 | -10.58 | 0.03153 | Oxidoreductase family |
| 16 | TRINITY_DN442519_c0_g1 | 0.00 | 0.00 | 172.08 | 40.10 | 55.85 | -10.57 | 0.00876 | Coffea canephora DH200=94 genomic scaffold |
| 17 | TRINITY_DN437344_c2_g1 | 0.00 | 0.00 | 375.23 | 79.16 | 126.20 | -10.56 | 0.02682 | TFL1 |
| 18 | TRINITY_DN445604_c0_g1 | 0.00 | 0.00 | 190.15 | 58.29 | 72.74 | -10.32 | 0.01208 | Putative protein E6 |
| 19 | TRINITY_DN438732_c0_g1 | 0.00 | 0.05 | 203.84 | 144.17 | 140.02 | -10.30 | 0.01033 | End binding protein 1C isoform 1 |
| 20 | TRINITY_DN436787_c4_g2 | 0.00 | 0.00 | 27.74 | 22.67 | 34.88 | -10.28 | 0.00831 | Fatty acid/sphingolipid desaturase |
| 21 | TRINITY_DN436541_c0_g1 | 0.00 | 0.00 | 172.83 | 12.78 | 31.52 | -10.25 | 0.02255 | Sulfotransferase |
| 22 | TRINITY_DN430929_c3_g1 | 0.00 | 0.00 | 39.49 | 15.22 | 82.23 | -10.23 | 0.02661 | Putative benzoate carboxyl methyltransferase |
| 23 | TRINITY_DN449002_c2_g1 | 0.00 | 0.00 | 14.54 | 17.02 | 14.11 | -10.03 | 0.00831 | Leucine-rich repeat protein kinase family protein isoform 1 |
| 24 | TRINITY_DN433129_c0_g3 | 0.00 | 0.00 | 19.65 | 51.10 | 9.66 | -9.97 | 0.00831 | DUF594 family protein |
| 25 | TRINITY_DN441888_c1_g2 | 0.00 | 0.00 | 31.98 | 13.24 | 21.72 | -9.87 | 0.00975 | Putative alpha-l-fucosidase |
| 26 | TRINITY_DN445462_c0_g1 | 0.00 | 0.00 | 18.94 | 9.06 | 13.69 | -9.83 | 0.00831 | Putative axoneme-associated protein mst101(2)-like |
| 27 | TRINITY_DN448863_c2_g1 | 0.00 | 0.17 | 74.18 | 52.40 | 57.82 | -9.83 | 0.03436 | Cyclin b1,5 isoform 1 |
| 28 | TRINITY_DN440010_c0_g3 | 0.20 | 0.00 | 163.02 | 100.07 | 203.67 | -9.74 | 0.02150 | Early nodulin-like protein 11 |
| 29 | TRINITY_DN433967_c0_g1 | 0.00 | 0.00 | 31.16 | 9.58 | 32.89 | -9.71 | 0.01425 | Putative glutaredoxin family protein |
| 30 | TRINITY_DN431440_c0_g1 | 0.00 | 0.00 | 33.37 | 31.23 | 18.69 | -9.67 | 0.00831 | Major facilitator superfamily protein |
| 31 | TRINITY_DN429524_c0_g2 | 0.00 | 0.00 | 13.80 | 29.16 | 111.83 | -9.67 | 0.03366 | Putative protein LURP-one-related 10-like |
| 32 | TRINITY_DN421435_c0_g1 | 0.00 | 0.00 | 631.57 | 778.98 | 281.62 | -9.61 | 0.00831 | Putative cytochrome P450 76M5 |
| 33 | TRINITY_DN449823_c1_g3 | 0.00 | 0.00 | 32.80 | 31.58 | 20.94 | -9.60 | 0.00831 | Coffea canephora DH200=94 genomic scaffold |
| 34 | TRINITY_DN439680_c0_g6 | 0.00 | 0.00 | 42.66 | 50.79 | 31.05 | -9.58 | 0.00831 | Rac-like GTP-binding protein ARAC7 |
| 35 | TRINITY_DN435630_c1_g1 | 0.00 | 0.00 | 53.19 | 21.68 | 28.87 | -9.58 | 0.00969 | Coffea canephora DH200=94 genomic scaffold |
| 36 | TRINITY_DN437970_c0_g1 | 0.10 | 0.00 | 58.15 | 27.49 | 41.18 | -9.57 | 0.04102 | Putative cyclin B1 |
| 37 | TRINITY_DN437130_c1_g1 | 0.00 | 0.00 | 36.12 | 11.62 | 15.18 | -9.56 | 0.01237 | Inflorescence meristem receptor-like kinase 2 isoform 1 |
| 38 | TRINITY_DN431447_c1_g1 | 0.00 | 0.00 | 32.85 | 59.82 | 18.01 | -9.54 | 0.00947 | Putative probable caffeine synthase 4-like |
| 39 | TRINITY_DN442369_c5_g2 | 0.00 | 0.00 | 53.50 | 10.64 | 23.20 | -9.54 | 0.01855 | Fasciclin-like arabinogalactan protein |
| 40 | TRINITY_DN436583_c0_g1 | 0.03 | 0.00 | 27.08 | 11.31 | 21.45 | -9.53 | 0.02922 | Mitotic-like cyclin 3B from |
| 41 | TRINITY_DN434313_c0_g3 | 0.00 | 0.00 | 27.96 | 14.49 | 26.52 | -9.52 | 0.00873 | Putative TPX2 protein family |
| 42 | TRINITY_DN426665_c0_g3 | 0.00 | 0.00 | 11.95 | 8.88 | 11.65 | -9.49 | 0.00831 | Laccase |
| 43 | TRINITY_DN435384_c0_g1 | 0.00 | 0.00 | 31.58 | 9.32 | 23.30 | -9.47 | 0.01380 | Cyclin family protein |
| 44 | TRINITY_DN440088_c0_g4 | 0.00 | 0.00 | 233.80 | 102.87 | 113.58 | -9.45 | 0.00947 | Putative peamaclein-like |
| 45 | TRINITY_DN447490_c0_g3 | 0.00 | 0.00 | 20.16 | 9.42 | 11.77 | -9.43 | 0.00888 | Kinesin-like protein |
| 46 | TRINITY_DN429845_c0_g2 | 0.00 | 0.00 | 178.70 | 129.28 | 113.96 | -9.40 | 0.00831 | Laccase |
| 47 | TRINITY_DN432607_c1_g1 | 0.00 | 0.00 | 57.60 | 42.73 | 37.96 | -9.37 | 0.00831 | Hydroxyproline-rich glycoprotein family protein |
| 48 | TRINITY_DN442867_c0_g1 | 0.00 | 0.00 | 50.14 | 32.79 | 8.70 | -9.36 | 0.01919 | Patatin |
| 49 | TRINITY_DN431526_c3_g1 | 0.00 | 0.00 | 41.99 | 29.01 | 107.47 | -9.36 | 0.01313 | Blue copper protein |
| 50 | TRINITY_DN435387_c0_g1 | 0.00 | 0.00 | 39.29 | 7.23 | 19.20 | -9.33 | 0.02094 | EXORDIUM like 1 |
| 51 | TRINITY_DN443586_c4_g5 | 0.00 | 0.00 | 122.27 | 68.15 | 16.16 | -9.31 | 0.02273 | Putative copper transporter |
| 52 | TRINITY_DN446037_c2_g2 | 0.00 | 0.00 | 164.64 | 33.30 | 88.45 | -9.27 | 0.01904 | Caffeic acid 3-O-methyltransferase |
| 53 | TRINITY_DN447121_c0_g1 | 0.00 | 0.00 | 5.56 | 5.15 | 6.68 | -9.26 | 0.00831 | Kinesin-like protein NACK2 |
| 54 | TRINITY_DN440550_c0_g1 | 0.00 | 0.00 | 55.67 | 15.68 | 40.14 | -9.24 | 0.01418 | Tetratricopeptide repeat-like superfamily protein isoform 1 |
| 55 | TRINITY_DN440357_c0_g1 | 0.00 | 0.00 | 13.20 | 7.40 | 8.53 | -9.21 | 0.00831 | Putative multicopper oxidase |
| 56 | TRINITY_DN434099_c0_g1 | 0.00 | 0.00 | 23.78 | 18.42 | 8.96 | -9.19 | 0.00881 | Putative probable auxin efflux carrier component 6 |
| 57 | TRINITY_DN446468_c0_g2 | 0.06 | 0.00 | 37.41 | 16.09 | 35.01 | -9.18 | 0.02288 | Putative Gb:AAF35421.1 |
| 58 | TRINITY_DN451342_c3_g2 | 0.00 | 0.00 | 24.32 | 19.54 | 21.03 | -9.17 | 0.00831 | DNA helicase |
| 59 | TRINITY_DN439387_c1_g1 | 0.00 | 0.00 | 13.53 | 11.62 | 6.54 | -9.16 | 0.00831 | MATE efflux family protein |
| 60 | TRINITY_DN437429_c1_g2 | 0.00 | 0.00 | 66.86 | 27.63 | 114.45 | -9.16 | 0.00866 | Putative vegetative cell wall protein gp1-like |
| 61 | TRINITY_DN442088_c1_g3 | 0.04 | 0.00 | 18.61 | 18.15 | 18.93 | -9.15 | 0.00881 | Myb family transcription factor family protein |
| 62 | TRINITY_DN428010_c1_g1 | 0.00 | 0.00 | 40.35 | 6.11 | 8.93 | -9.14 | 0.02571 | Lipoxygenase |
| 63 | TRINITY_DN435856_c0_g1 | 0.00 | 0.00 | 24.90 | 16.49 | 24.24 | -9.12 | 0.00831 | Coffea canephora DH200=94 genomic scaffold |
| 64 | TRINITY_DN428756_c0_g1 | 0.00 | 0.00 | 122.13 | 20.03 | 19.68 | -9.10 | 0.02697 | Non-specific lipid-transfer protein |
| 65 | TRINITY_DN429116_c2_g2 | 0.00 | 0.00 | 63.98 | 26.10 | 55.57 | -9.04 | 0.00923 | Ankyrin repeat family protein |
| 66 | TRINITY_DN436389_c2_g2 | 11.09 | 0.00 | 322.05 | 435.65 | 187.79 | -9.04 | 0.01561 | MYB transcription factor R2R3-like protein |
| 67 | TRINITY_DN430302_c1_g1 | 0.00 | 0.00 | 49.99 | 35.24 | 33.62 | -9.02 | 0.00831 | Putative CLAVATA3/ESR (CLE)-related protein 46 |
| 68 | TRINITY_DN426923_c6_g2 | 0.00 | 0.00 | 41.95 | 43.32 | 62.84 | -9.02 | 0.00831 | Putative cytochrome P450 |
| 69 | TRINITY_DN441008_c0_g1 | 0.00 | 0.00 | 13.99 | 6.10 | 11.01 | -9.01 | 0.00873 | Calmodulin-binding family protein |
| 70 | TRINITY_DN432517_c1_g1 | 0.00 | 0.00 | 24.75 | 13.84 | 24.47 | -9.01 | 0.00831 | Putative polygalacturonase At1g48100 |
| 71 | TRINITY_DN439238_c0_g1 | 0.00 | 0.00 | 40.01 | 39.17 | 57.14 | -9.00 | 0.00831 | PREDICTED: putative late blight resistance protein homolog R1B-16 |
| 72 | TRINITY_DN443376_c0_g1 | 0.00 | 0.00 | 9.55 | 5.78 | 6.48 | -8.98 | 0.00831 | Putative aspartyl-tRNA synthetase |
| 73 | TRINITY_DN443478_c1_g1 | 0.10 | 0.00 | 41.92 | 27.26 | 51.55 | -8.97 | 0.01547 | Mitotic spindle checkpoint MAD2-like protein |
| 74 | TRINITY_DN438982_c0_g1 | 0.00 | 0.00 | 13.67 | 8.28 | 7.77 | -8.94 | 0.00831 | Coffea canephora DH200=94 genomic scaffold, scaffold_12 |
| 75 | TRINITY_DN440550_c1_g2 | 0.00 | 0.00 | 15.82 | 14.47 | 14.50 | -8.94 | 0.00831 | Tetratricopeptide repeat-like superfamily protein isoform 1 |
| 76 | TRINITY_DN441791_c0_g1 | 0.00 | 0.00 | 8.90 | 2.34 | 4.14 | -8.91 | 0.01367 | Kinesin-like protein |
| 77 | TRINITY_DN438742_c0_g1 | 0.00 | 0.00 | 10.33 | 10.13 | 14.72 | -8.91 | 0.00831 | PHD finger family protein |
| 78 | TRINITY_DN445073_c0_g1 | 0.00 | 0.00 | 190.65 | 26.03 | 42.53 | -8.91 | 0.02550 | TFL1 |
| 79 | TRINITY_DN436462_c2_g1 | 0.00 | 0.00 | 15.85 | 4.99 | 13.98 | -8.90 | 0.01085 | Pectinacetylesterase family protein |
| 80 | TRINITY_DN436536_c0_g1 | 0.00 | 0.00 | 9.73 | 17.01 | 21.79 | -8.90 | 0.00831 | Putative GDSL esterase/lipase At1g54790 isoform X1 |
| 81 | TRINITY_DN441415_c1_g1 | 0.00 | 0.00 | 719.09 | 691.91 | 1100.77 | -8.90 | 0.00873 | Amino acid permease 2 isoform 1 |
| 82 | TRINITY_DN441041_c2_g2 | 0.15 | 0.00 | 39.06 | 30.40 | 44.77 | -8.89 | 0.01760 | Kinesin-like protein |
| 83 | TRINITY_DN437601_c0_g1 | 0.00 | 0.00 | 9.70 | 16.54 | 7.60 | -8.86 | 0.00831 | Transducin family protein |
| 84 | TRINITY_DN433832_c1_g1 | 0.00 | 0.00 | 136.44 | 20.03 | 144.35 | -8.85 | 0.00831 | Predicted protein |
| 85 | TRINITY_DN440102_c0_g6 | 0.00 | 0.00 | 67.02 | 151.92 | 56.65 | -8.85 | 0.00831 | ACD1-like |
| 86 | TRINITY_DN444555_c0_g2 | 0.04 | 0.00 | 14.10 | 7.30 | 8.94 | -8.84 | 0.02506 | Kinesin-like protein |
| 87 | TRINITY_DN443967_c2_g1 | 0.00 | 0.00 | 4.59 | 11.45 | 1.30 | -8.82 | 0.02134 | Putative E3 ubiquitin-protein ligase LIN-1 |
| 88 | TRINITY_DN440590_c0_g1 | 0.00 | 0.00 | 9.13 | 3.04 | 4.51 | -8.81 | 0.01085 | Leucine-rich repeat receptor-like protein kinase PXL1 |
| 89 | TRINITY_DN441762_c6_g2 | 1.75 | 0.00 | 72.76 | 98.48 | 76.09 | -8.81 | 0.04531 | Putative polygalacturonase-1 non-catalytic subunit beta |
| 90 | TRINITY_DN433890_c0_g1 | 0.00 | 0.00 | 74.46 | 36.08 | 77.24 | -8.80 | 0.00903 | Histone superfamily protein |
| 91 | TRINITY_DN442985_c0_g1 | 0.04 | 0.00 | 11.32 | 12.52 | 16.24 | -8.79 | 0.01085 | Cytochrome P450 78A3p family protein |
| 92 | TRINITY_DN440654_c0_g2 | 0.00 | 0.00 | 12.57 | 6.16 | 11.27 | -8.78 | 0.00873 | Portal 56 |
| 93 | TRINITY_DN430441_c3_g3 | 0.00 | 0.00 | 273.11 | 182.18 | 231.44 | -8.78 | 0.00831 | High mobility group family protein |
| 94 | TRINITY_DN428337_c0_g1 | 0.00 | 0.00 | 10.51 | 10.19 | 7.33 | -8.76 | 0.00831 | Flavin-containing monooxygenase |
| 95 | TRINITY_DN434566_c3_g1 | 0.00 | 0.00 | 11.02 | 14.09 | 15.25 | -8.76 | 0.00831 | Putative zinc finger protein |
| 96 | TRINITY_DN431205_c0_g1 | 0.00 | 0.00 | 24.03 | 17.86 | 21.52 | -8.76 | 0.00831 | Coffea canephora DH200=94 genomic scaffold |
| 97 | TRINITY_DN429779_c1_g1 | 0.00 | 0.00 | 58.85 | 23.61 | 49.49 | -8.76 | 0.00976 | At1g31335 |
| 98 | TRINITY_DN439238_c0_g2 | 0.00 | 0.00 | 48.60 | 18.89 | 25.92 | -8.73 | 0.00947 | Jacalin-related lectin 3 |
| 99 | TRINITY_DN442303_c1_g3 | 0.00 | 0.00 | 18.16 | 5.98 | 30.21 | -8.73 | 0.01085 | Cyclin family protein |
| 100 | TRINITY_DN443823_c0_g4 | 0.34 | 0.00 | 104.75 | 70.92 | 64.68 | -8.73 | 0.01848 | Predicted WEB family protein At2g40480 |

**Table 5S. Upregulated transcripts in common (Top 100) between resistant and susceptible genotypes when compared to water.**

| **Transcript ID** | **Description** |
| --- | --- |
| TRINITY_DN373295_c0_g2 | Predicted protein |
| TRINITY_DN425980_c1_g2 | Cell wall protein |
| TRINITY_DN426185_c1_g2 | GPI anchored serine-rich protein |
| TRINITY_DN426651_c2_g1 | Alkaline proteinase |
| TRINITY_DN431010_c0_g1 | T1.1 protein |
| TRINITY_DN436844_c0_g1 | Predicted protein |
| TRINITY_DN437425_c6_g3 | Peptidyl-prolyl cis-trans isomerase |
| TRINITY_DN439596_c1_g1 | Putative growth factor independence |
| TRINITY_DN442555_c0_g1 | Putative inactive poly [ADP-ribose] polymerase SRO2 |
| TRINITY_DN447378_c2_g1 | Glycosyltransferase |
| TRINITY_DN463178_c0_g1 | Cutinase |

**Table 6S. Downregulated transcripts in common (Top 100) between resistant and susceptible genotypes when compared to water.**

| **Transcript ID** | **Description** |
| --- | --- |
| TRINITY_DN428010_c1_g1 | Lipoxygenase |
| TRINITY_DN429116_c2_g2 | Ankyrin repeat family protein |
| TRINITY_DN429524_c0_g2 | Putative protein LURP-one-related 10-like |
| TRINITY_DN429852_c0_g1 | Sulfotransferase |
| TRINITY_DN432222_c0_g2 | 4-coumarate:CoA ligase 3 |
| TRINITY_DN432607_c1_g1 | Hydroxyproline-rich glycoprotein family protein |
| TRINITY_DN433129_c0_g3 | DUF594 family protein |
| TRINITY_DN433832_c1_g1 | Predicted protein |
| TRINITY_DN434313_c0_g3 | Putative TPX2 protein family |
| TRINITY_DN435384_c0_g1 | Cyclin family protein |
| TRINITY_DN436787_c4_g2 | Fatty acid/sphingolipid desaturase |
| TRINITY_DN437970_c0_g1 | Putative cyclin B1 |
| TRINITY_DN438303_c0_g1 | Purple acid phosphatase |
| TRINITY_DN438742_c0_g1 | PHD finger family protein |
| TRINITY_DN440088_c0_g4 | Putative peamaclein-like |
| TRINITY_DN440654_c0_g2 | Portal 56 |
| TRINITY_DN441216_c1_g2 | Leucoanthocyanidin reductase |
| TRINITY_DN441415_c1_g1 | Amino acid permease 2 isoform 1 |
| TRINITY_DN441888_c1_g2 | Alpha-l-fucosidase, putative |
| TRINITY_DN442303_c1_g3 | Cyclin family protein |
| TRINITY_DN442867_c0_g1 | Patatin |
| TRINITY_DN444245_c2_g3 | Reticulon-like protein |
| TRINITY_DN445462_c0_g1 | Putative axoneme-associated protein mst101(2)-like |
| TRINITY_DN446037_c2_g2 | Caffeic acid 3-O-methyltransferase |
| TRINITY_DN447490_c0_g3 | Kinesin-like protein |


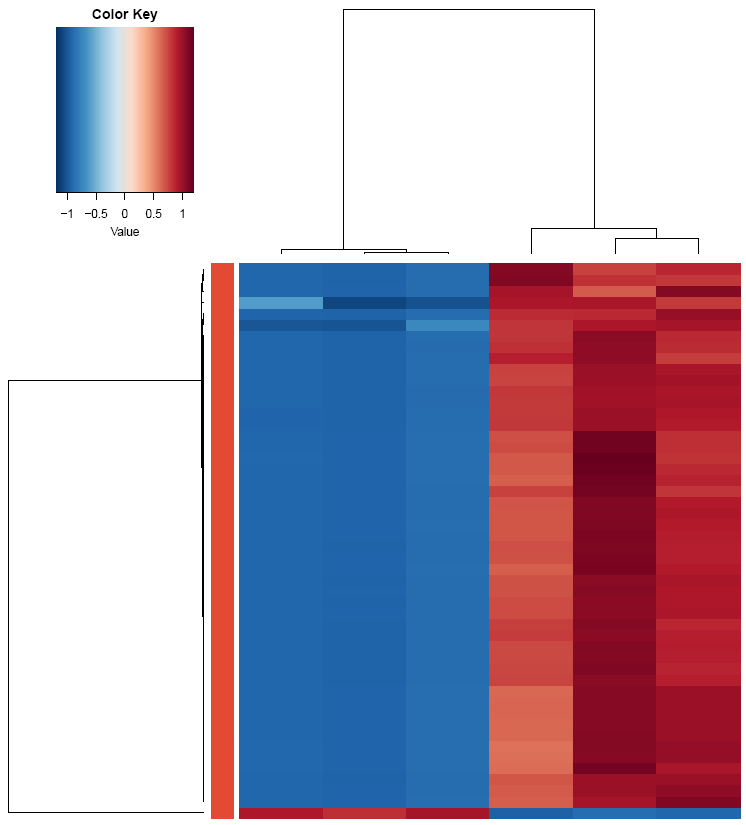


**Figure 1S.** Top 50 differentially expressed genes between nickel-treated resistant and water-treated Acer *rubrum* genotypes based on logFC. The red colour represents an upregulation and blue downregulation

Water_ctrl_17

Water_ctrl_16

Water_ctrl_60

Total_Res_28

Total_Res_5

Total_Res_52

TRINITY_DN450340_c1_g4 ** CYP81Q32 n=1 Tax=

TRINITY_DN439596_c1_g1 ** Growth factor independence

TRINITY_DN447378_c2_g1 ** Glycosyltransferase,

TRINITY_DN442555_c0_g1 ** PREDICTED: probable

TRINITY_DN441929_c1_g4 ** PREDICTED: putative

TRINITY_DN420778_c2_g4 ** Proteinase T n=1 Tax=

TRINITY_DN402821_c0_g1 ** Glycoside hydrolase

TRINITY_DN428667_c1_g1 ** GRF domain class

TRINITY_DN433554_c0_g1 ** 17.5 kd heat shock

TRINITY_DN440872_c0_g1 ** Trypsin−like protease

TRINITY_DN426651_c1_g1 ** Alkaline proteinase

TRINITY_DN425833_c0_g1 ** Predicted protein n=

TRINITY_DN426651_c2_g1 ** Alkaline proteinase

TRINITY_DN408982_c0_g2 ** Putative fad fmn−containing

TRINITY_DN407838_c0_g1 ** Zinc carboxypeptidase

TRINITY_DN433234_c4_g8 ** 60S ribosomal protein

TRINITY_DN449832_c0_g1 ** Glycoside hydrolase

TRINITY_DN419762_c0_g1 ** Beta−glucosidase n=

TRINITY_DN417245_c0_g3 ** Predicted protein n=

TRINITY_DN436844_c0_g1 ** Predicted protein n=

TRINITY_DN418791_c0_g1 ** Predicted protein n=

TRINITY_DN435263_c4_g1 ** 40S ribosomal protein

TRINITY_DN431808_c3_g3 ** Fusarium graminearum

TRINITY_DN430296_c0_g1 ** Predicted protein n=

TRINITY_DN373295_c0_g2 ** Predicted protein n=

TRINITY_DN440532_c2_g1 ** Guanine nucleotide

TRINITY_DN436706_c1_g2 ** Probable ribosomal −

TRINITY_DN434934_c0_g2 ** Ribosomal protein S29

TRINITY_DN431192_c3_g2 ** Ribosomal protein L28,

TRINITY_DN437630_c5_g2 ** Ribosomal protein S30

TRINITY_DN417293_c1_g1 ** Peptide hydrolase n=

TRINITY_DN422756_c0_g1 ** Elongation factor 1−

TRINITY_DN396774_c0_g1 ** Glycoside hydrolase

TRINITY_DN423641_c2_g3 ** 1,3−beta−glucan synthase

TRINITY_DN413617_c0_g1 ** Alcohol oxidase n=

TRINITY_DN422735_c0_g1 ** Ribosomal L28e

TRINITY_DN433729_c2_g3 ** Carboxypeptidase A family

TRINITY_DN463178_c0_g1 ** Cutinase n=3 Tax=Trichoderma

TRINITY_DN439689_c8_g2 ** Elongation factor 2

TRINITY_DN427051_c3_g1 ** Alpha−1,4 glucan phosphorylase

TRINITY_DN425700_c1_g1 ** Glycerol kinase−like

TRINITY_DN422594_c0_g1 ** Predicted protein n=

TRINITY_DN440752_c0_g1 ** Methyltransferase domain−

TRINITY_DN438386_c0_g1 ** Inorganic pyrophosphatase

TRINITY_DN429713_c4_g1 ** Fructose bisphosphate

TRINITY_DN423546_c0_g3 ** Fusarium graminearum

TRINITY_DN432905_c4_g3 ** Transaldolase n=4

TRINITY_DN418601_c0_g2 ** Predicted protein n=

TRINITY_DN425242_c0_g1 ** Predicted protein n=

TRINITY_DN438303_c0_g1 ** Purple acid phosphatase


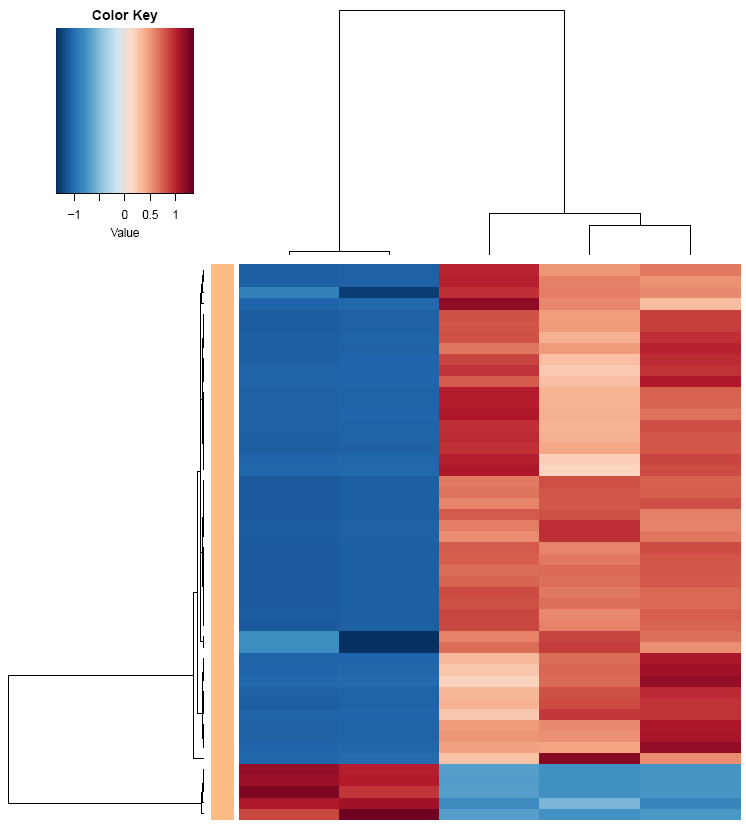


**Figure 2S.** Top 50 differentially expressed genes between nickel-treated resistant and susceptible *Acer rubrum* genotypes based on logFC. The red colour represents an upregulation and blue downregulation.

Total_22

Total_43

Total_Res_52

Total_Res_28

Total_Res_5

TRINITY_DN375089_c0_g1 * Ornithine monooxygenase

TRINITY_DN429308_c2_g4 * F5M15.26 n=1 Tax=

TRINITY_DN445296_c1_g4 * 60S ribosomal protein

TRINITY_DN446020_c0_g1 * S−adenosylmethionine−

TRINITY_DN450475_c1_g1 * PREDICTED: F−box/

TRINITY_DN431953_c5_g1 * hypothetical protein

TRINITY_DN330872_c0_g2 * Fusarium graminearum,

TRINITY_DN445835_c1_g2 * PREDICTED: cytochrome

TRINITY_DN416544_c0_g1 * Predicted protein n=

TRINITY_DN329922_c0_g1 * Predicted protein n=

TRINITY_DN423469_c0_g1 * Putative short chain

TRINITY_DN430442_c0_g1 * Predicted protein n=

TRINITY_DN419449_c0_g2 * Serine/threonine−protein

NA * NA

NA * NA

TRINITY_DN325824_c0_g1 * Coatomer subunit alpha

TRINITY_DN362203_c0_g1 * DUF300−domain−containing

TRINITY_DN403025_c0_g2 * Predicted protein n=

TRINITY_DN314982_c1_g1 * Predicted protein n=

NA * NA

TRINITY_DN436216_c0_g1 * PREDICTED: putative

TRINITY_DN444610_c0_g5 * PREDICTED: putative

TRINITY_DN418021_c0_g1 * Glutathione S−transferase

NA * NA

TRINITY_DN431526_c3_g1 * Blue copper protein

TRINITY_DN402230_c1_g1 * Carbon catabolite repressor

NA * NA

TRINITY_DN438325_c0_g2 * PREDICTED: NADH−

TRINITY_DN431953_c4_g1 * Formin−like

TRINITY_DN377100_c0_g1 * Predicted protein n= protein

TRINITY_DN441905_c0_g10 * PREDICTED: protein

TRINITY_DN411075_c0_g1 * Defective in cullin neddylation

TRINITY_DN431804_c3_g1 * Caffeic acid 3−O−methyltransferase

TRINITY_DN431493_c1_g1 * Ankyrin repeat−containing

NA * NA

TRINITY_DN431447_c1_g1 * PREDICTED: probable

TRINITY_DN431440_c0_g1 * Major facilitator superfamily

TRINITY_DN433451_c3_g1 * PREDICTED: ankyrin

TRINITY_DN421435_c0_g1 * PREDICTED: cytochrome

NA * NA

TRINITY_DN449745_c1_g2 * Tetratricopeptide repeat−

TRINITY_DN370817_c0_g2 * Predicted protein n=

TRINITY_DN427744_c1_g1 * Predicted protein n=

TRINITY_DN440779_c1_g3 * Anthocyanidin

TRINITY_DN433899_c1_g1 * sugar transporter n= reductase

TRINITY_DN434380_c0_g1 * Aquaporin NIP6.1 family

TRINITY_DN429635_c5_g1 * Peroxisomal ascorbate

TRINITY_DN66483_c0_g2 * Translation initiation factor

NA * NA

TRINITY_DN15726_c0_g1 * membrane protein n=


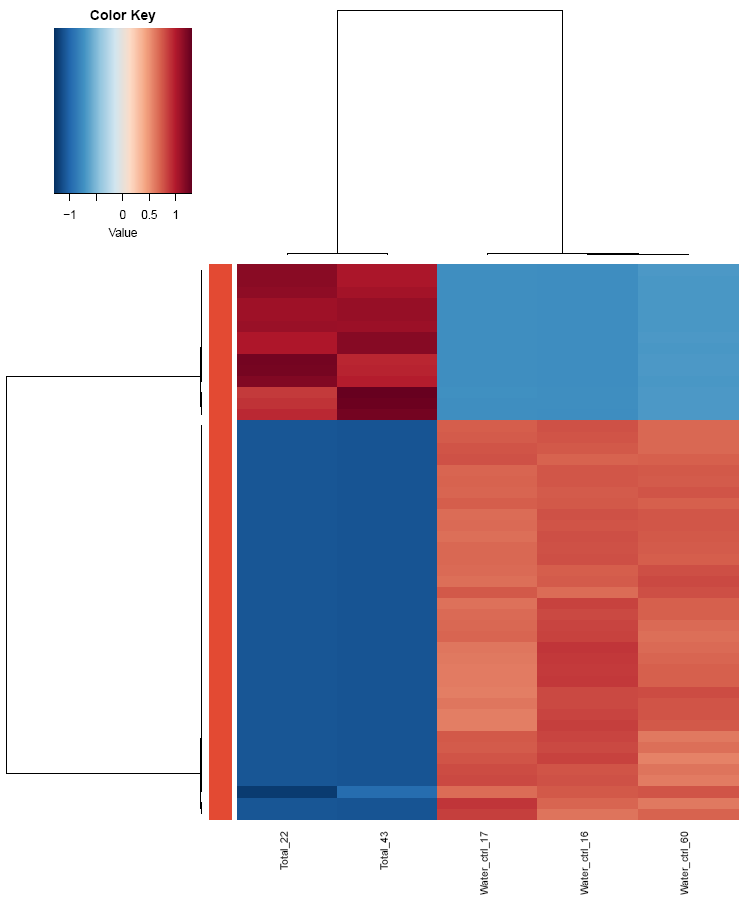


**Figure 2S.** Top 50 differentially expressed genes between nickel-treated susceptible and water-treated Acer *rubrum* genotypes based on logFC. The red colour represents an upregulation and blue downregulation.

Total_43

Total_22

Water_ctrl_16

Water_ctrl_60

Water_ctrl_17

TRINITY_DN459004_c0_g1 ** RpL31_0 protein (Fragment)

TRINITY_DN432097_c0_g1 ** Chloride channel protein
TRINITY_DN426807_c0_g1 ** Albugo candida WGS

TRINITY_DN445195_c1_g1 ** PREDICTED: cucumisin−

NA ** NA

TRINITY_DN435926_c1_g6 ** DCD domain protein

TRINITY_DN451117_c0_g4 ** Kinase−like protein

TRINITY_DN451117_c1_g2 ** Kinase, putative n=

TRINITY_DN436977_c0_g1 ** Cyclin p4 n=1 Tax=

TRINITY_DN375151_c0_g1 ** 60S ribosomal protein

TRINITY_DN446102_c0_g1 ** Glycine−rich protein

TRINITY_DN431010_c0_g1 ** T1.1 protein n=4 Tax=

TRINITY_DN446442_c1_g1 ** Non−symbiotic hemoglobin

TRINITY_DN428750_c1_g1 ** Tonoplast intrinsic protein,

TRINITY_DN449002_c2_g1 ** Leucine−rich repeat

TRINITY_DN440428_c2_g2 ** AMP dependent CoA

TRINITY_DN443586_c4_g3 ** Copper transporter

TRINITY_DN440779_c1_g3 ** Anthocyanidin reductase

TRINITY_DN429845_c0_g2 ** Laccase n=1 Tax=Jatropha

TRINITY_DN430221_c0_g1 ** Limonene synthase

TRINITY_DN430737_c1_g1 ** Cupredoxin superfamily

TRINITY_DN432222_c0_g2 ** 4−coumarate:CoA

TRINITY_DN426665_c0_g3 ** Laccase n=3 Tax=Citrus

TRINITY_DN444245_c2_g3 ** Reticulon−like protein

TRINITY_DN445462_c0_g1 ** PREDICTED: axoneme−

TRINITY_DN451342_c3_g2 ** DNA helicase n=2 Tax=

TRINITY_DN441216_c1_g2 ** Leucoanthocyanidin

TRINITY_DN447121_c0_g1 ** Kinesin−like protein

TRINITY_DN436787_c4_g2 ** Fatty acid/sphingolipid

TRINITY_DN438303_c0_g1 ** Purple acid phosphatase

TRINITY_DN440357_c0_g1 ** Multicopper oxidase

TRINITY_DN449823_c1_g3 ** Coffea canephora DH20

TRINITY_DN432607_c1_g1 ** Hydroxyproline−rich =,

TRINITY_DN433129_c0_g3 ** DUF594 family protein

TRINITY_DN440088_c0_g4 ** PREDICTED: peamaclein−

TRINITY_DN445604_c0_g1 ** PREDICTED: protein

TRINITY_DN447490_c0_g3 ** Kinesin−like protein

TRINITY_DN435630_c1_g1 ** Coffea canephora DH200=

TRINITY_DN434313_c0_g3 ** TPX2 protein family,

TRINITY_DN442519_c0_g1 ** Coffea canephora DH200=

TRINITY_DN437429_c1_g2 ** PREDICTED: vegetative

TRINITY_DN441888_c1_g2 ** Alpha−l−fucosidase

TRINITY_DN439387_c1_g1 ** MATE efflux family,

TRINITY_DN431440_c0_g1 ** Major facilitator superfamily

TRINITY_DN434099_c0_g1 ** PREDICTED: probable

TRINITY_DN439680_c0_g6 ** Rac−like GTP−binding

TRINITY_DN421435_c0_g1 ** PREDICTED: cytochrome

TRINITY_DN438732_c0_g1 ** End binding protein

TRINITY_DN431447_c1_g1 ** PREDICTED: probable

TRINITY_DN434133_c0_g3 ** Coffea canephora DH200=
